# Supplementary material for: A hierarchical 3D-motion learning framework for animal spontaneous behavior mapping
Source: Nat Commun. 2021 May 13;12:2784. doi: 10.1038/s41467-021-22970-y (PMC8119960; doi:10.1038/s41467-021-22970-y)
Supplement: Supplementary file 1 — Supplementary Information [file 41467_2021_22970_MOESM1_ESM.pdf]

Supplementary Figures

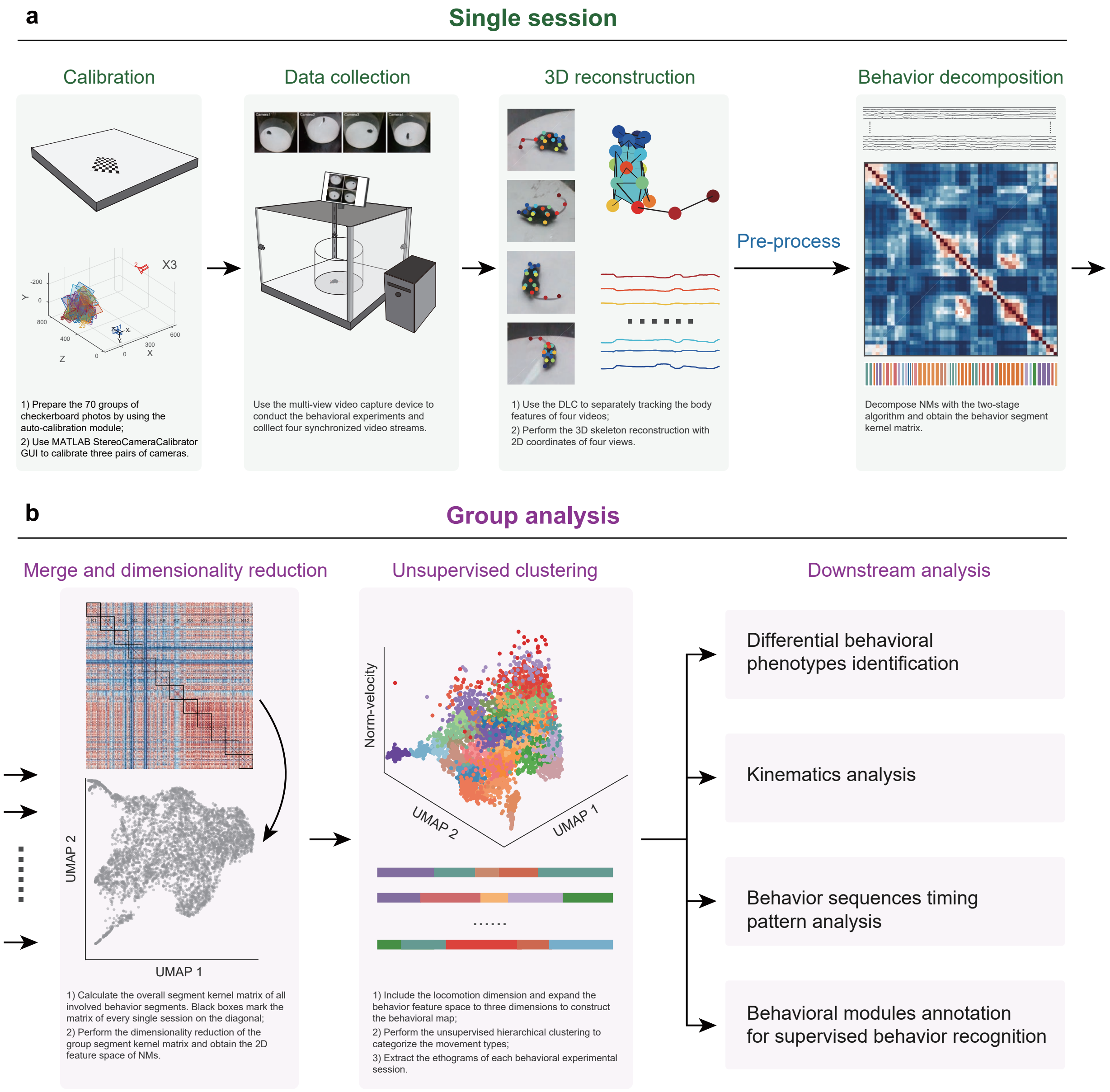

Supplementary Fig. 1 | The workflow of the hierarchical 3D-motion learning framework. a Four main steps for a single experimental session: 1) Calibration (related to Supplementary Methods "The calibration of 3D motion capture system"). Using the auto-calibration module to quickly prepare 70 groups of checkerboard images from various angles and positions for calibration and using the MATLAB StereoCameraCalibrator GUI to calculate the calibration parameters of the three pairs of cameras. This step is necessary only when the calibration parameters are unknown, or the cameras have been moved. 2) Data collection (related to Supplementary Methods Animals, behavioral experiments and behavioral data collection). Setting up the behavioral apparatus and preparing the animal and then using the multi-view video capture device to collect the synchronous behavioral videos. 3) 3D reconstruction (related to Supplementary Methods 3D pose reconstruction). Using the DLC pre-trained

model to predict the animal's 16 body-part 2D coordinates from the four separate videos, then performing the 3D skeleton reconstruction with the 2D coordinates from four views to obtain the animal's postural time-series. 4) Behavior decomposition (related to Supplementary Methods Behavior decomposition). Performing the two-stage behavior decomposition on the pre-processed postural time-series. This step discovers the behavioral modules based on the optimal movement segmentation. Finally, these behavioral segments are aligned using the DTAK metric to construct the segment kernel matrix representing their similarity. **b** Group analysis based on specific biological questions. 1) Merge and dimensionality reduction (related to Supplementary Methods: Group segment kernel matrix and low dimensional embedding). According to experimental grouping, single session segment kernel matrices are merged into a group segment kernel matrix. To visualize the informative structure of the behavioral modules involved, we used dimensionality reduction to transform the group segment kernel matrix into a 2D space. 2) Unsupervised clustering (related to Supplementary Methods: Unsupervised clustering). Constructing the behavioral map by combining the NM space with the locomotion dimension, then using the unsupervised clustering algorithm to categorize the movement sequence into distinct types. After clustering, ethograms can be constructed by associating the behavioral labels with their original segments. 3) Downstream analysis. After obtaining each session's ethogram, the downstream quantitative analysis can be conducted according to experimental grouping, recording stage, and other conditions to answer biological questions from behavioral aspects.

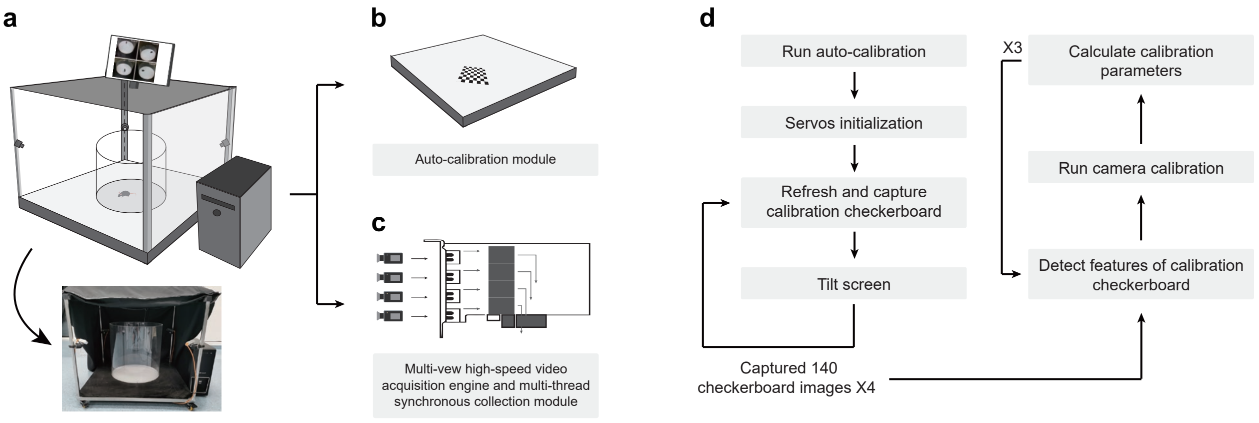

Supplementary Fig. 2 | Illustration of the multi-view video capture device and the workflow of the auto-calibration module. a Schematic of the multi-view video capture device. The support framework is a 90 × 90 × 75 cm^3 movable stainless steel shelf, on which cameras, behavioral apparatus, calibration modules, and background lighting are mounted. A shielding curtain can be added per experimental requirements. **b** The auto-calibration module is designed for efficient camera calibration in 3D and is composed of an LCD screen for displaying the checkerboard and a control unit used to tilt the screen. To collect images of the checkerboard pattern at different orientations relative to the cameras, the calibration program controls the screen to rotate and translate the checkerboard pattern at different tilt angles. With this auto-calibration module, the checkerboard images can be captured in one minute. **c** The multi-view video acquisition module. Four video streams, one per camera, are input to the PCI-E USB-3.0 data acquisition card (expanded bandwidth). The acquisition program then uses multi-thread acquisition to ensure frame synchronization. **d** The two-part workflow of the auto-calibration program. The first part, shown on the left, automatically collects a variety of checkerboard patterns for each camera (70). Right, the calibration process, which is based on the MATLAB StereoCameraCalibrator GUI.

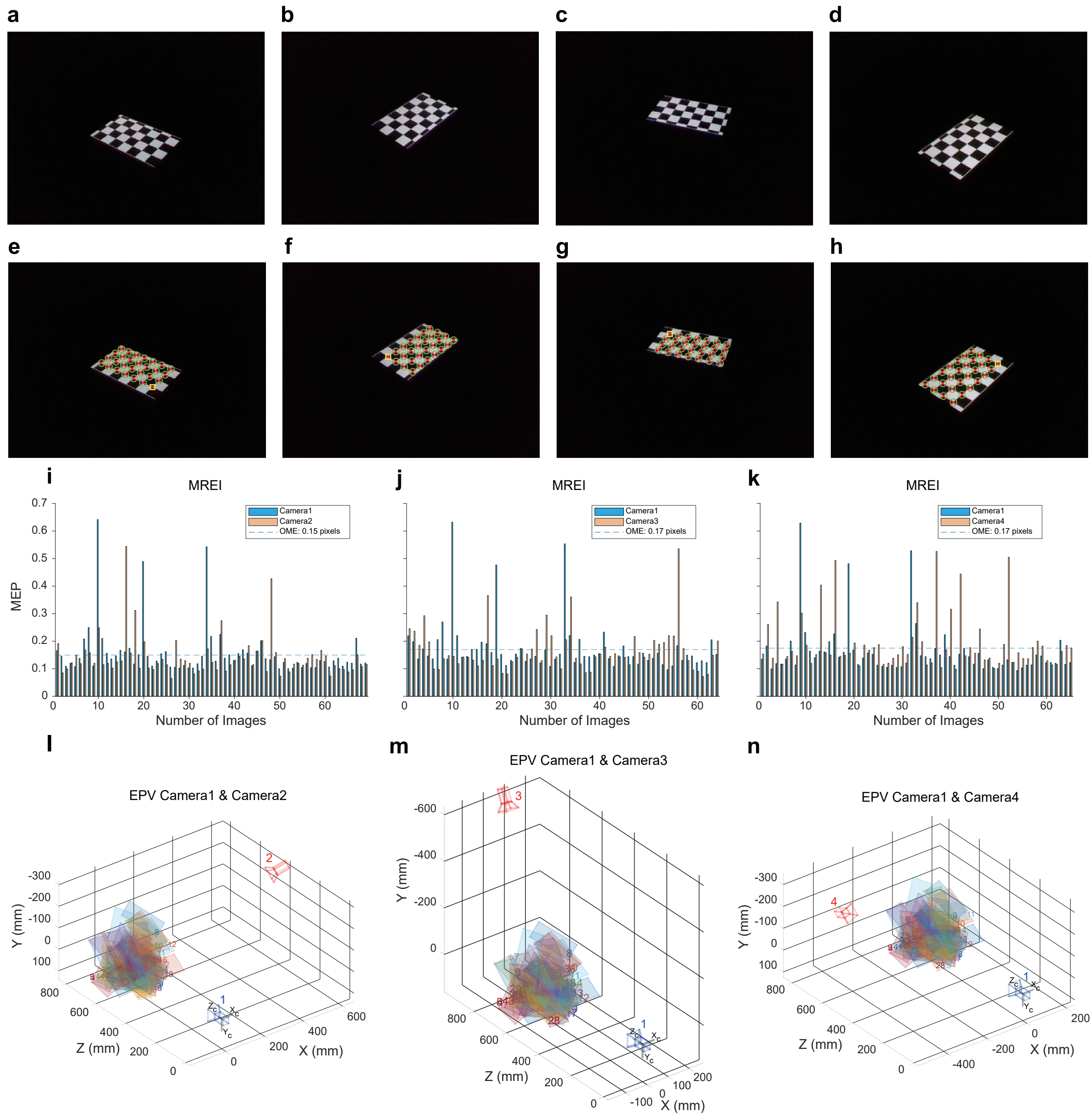

Supplementary Fig. 3 | Calibration of cameras results. a-d one of 70 checkerboard images of each camera in four different placements. All of the checkerboards are fully captured in four different cameras placements with dark light. We use a 10-inch tablet to display checkerboard to calibrate four cameras, which could freely adjust the brightness and get appropriate brightness easily to keep the checkerboard clear enough. **e-h** the result of grid detection with MATLAB's StereoCameraCalibrator GUI corresponding to **a-c** and **d** images. The corner recognition of the checkerboard is located on the corner of the black squares. **i-k** mean reprojection of error per image (MREI). MREI gives the quantization level of calibration error between cameras and checkerboards, where the mean error in pixels (MEP) represents the pixel errors in each camera-checkerboard pair and overall mean error (OME) gives a whole estimation of pixel errors. **l-n** extrinsic parameters visualization (EPV) of the cameras. EPV shows the relative positions of two cameras and checkerboards in 3D space. If EPV is different from real relative positions, we should calibrate two cameras again until they are coincident.

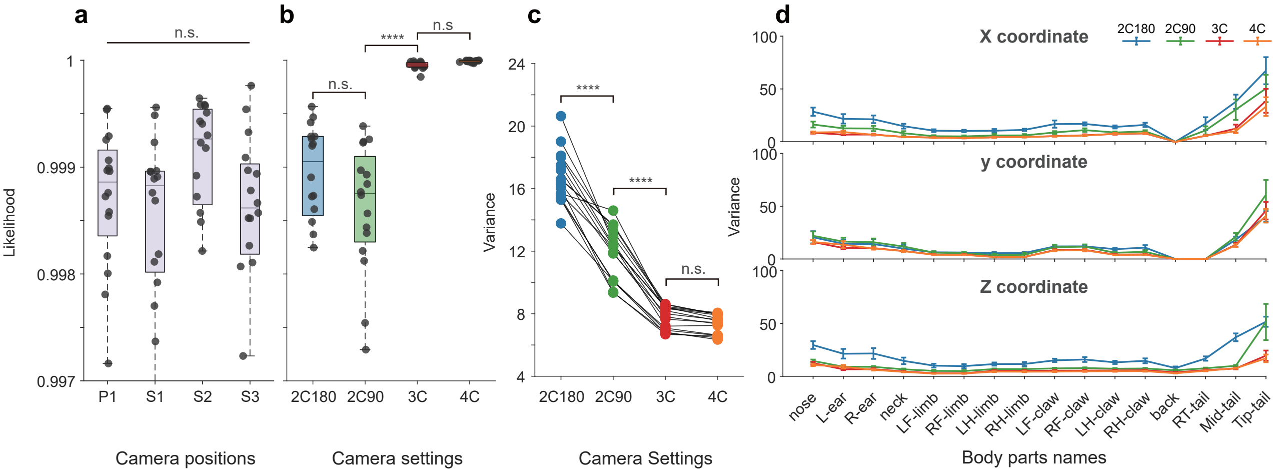

Supplementary Fig. 4 | Evaluation of 3D Reconstruction Quality with Different Camera Settings. **a** The likelihoods of the DLC pose estimations of four camera positions. P1, primary camera 1, S1, secondary camera 1, S2, secondary camera 2, S3, secondary camera 3. Each point on the boxplot represents the mean likelihood of each test recording, which is calculated by firstly averaging the likelihoods of all the body parts per frame then averaging them across all frames. The likelihoods show no significant differences among these cameras (One-sided Kruskal-Wallis test followed by Dunn's multiple comparisons test, $p = 0.1339$, $n = 16$). **b** The likelihoods of the 3D reconstructions of different camera groupings. 2C180, two cameras are placed in opposite directions. 2C90, two cameras are positioned in orthogonal directions. 3C, three cameras. 4C, four cameras. In the camera groupings 2C180 and 2C90, each point on the boxplot is calculated by firstly specifically averaging the likelihoods of two paired body parts for calibration, then averaging all 16 paired averaged likelihoods per frame and finally averaging them across all frames. In the camera groupings of 3C and 4C, each point on the boxplot is calculated by firstly specifically averaging the first two maximum likelihoods of paired body parts for calibration from all the three or four points, then averaging all 16 paired averaged maximum likelihoods per frame, and finally averaging them across all frames. (One-way ANOVA followed by Tukey's multiple comparisons test, ****$P < 0.0001$, $q = 10.62$, $DF = 60$; $n = 16$). **c** The variances of the behavioral trajectories captured by different camera groupings. Each point on the plot is calculated by firstly computing the variances of each body part's trajectory in the X, Y, Z axis, then averaging them across X, Y, Z axis, and finally averaging them across 16 body parts. (One-way ANOVA followed by Tukey's multiple comparisons test, ****2C180 V.S. 2C90, $P < 0.0001$, $q = 15.15$, $DF = 60$; ****2C90 V.S. 3C, $P < 0.0001$, $q = 13.14$, $DF = 60$; $n = 16$). **d** The variances of each body part in X, Y, Z coordinates of varying camera groupings, shown by body parts separately and presented as mean ± standard deviation (SD). The variances of each body part are calculated by firstly computing the variances of each body part's trajectory in X, Y, Z axis then averaging them across X, Y, Z axis. In box plots, the lower and upper edges of the box are the 25th and 75th percentiles of the values, the central marks indicate the median, the lower and upper whiskers are the minima, maxima values.

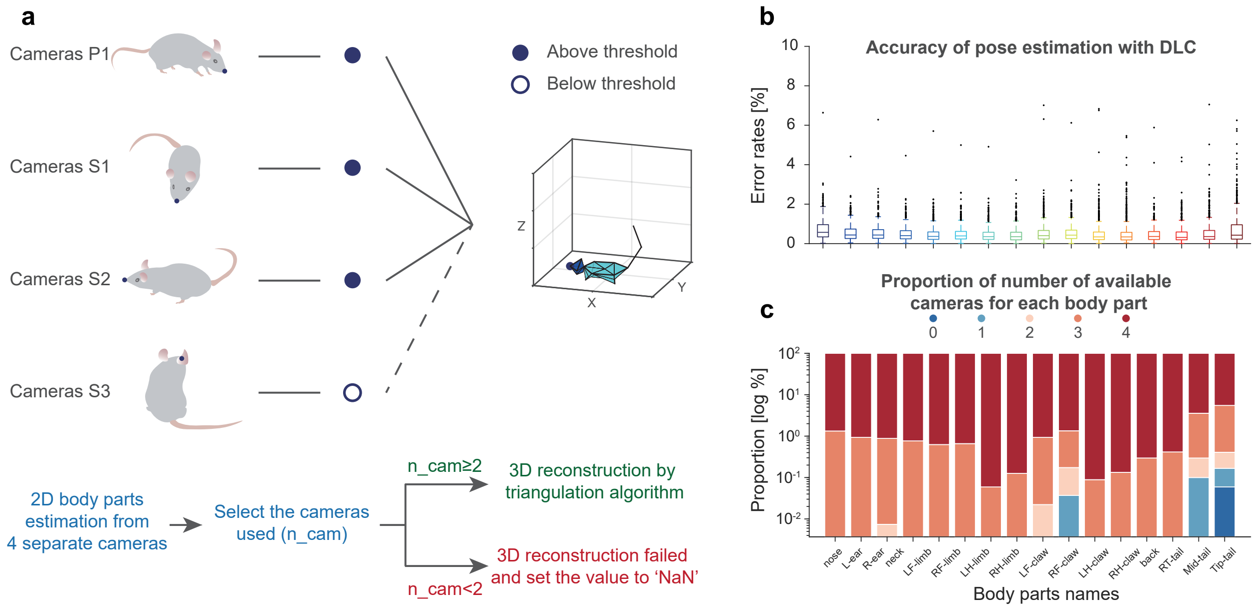

Supplementary Fig. 5 | 3D reconstruction process and reliability evaluation of the occluded body parts. a The workflow of 3D reconstruction of a single body part: 1) estimate the two-dimensional coordinates of the animal's body part from four cameras; 2) select the cameras to be used for reconstruction by thresholding the likelihood of the estimated body part; 3) determine whether the number of cameras available meets the reconstruction requirements (2 or more); and 4) if 2 or more, reconstruct the 3D coordinate of the body part. Otherwise, the 3D reconstruction fails due to the occlusion. P1, primary camera. S1, first secondary camera. S2, second secondary camera. S3, third secondary camera. **b** The errors in 2D body-part estimations versus ground truth. The error rates are shown for each body part separately in the boxplot and averaged 0.534 ± 0.005%. In box plots, the lower and upper edges of the box are the 25th and 75th percentiles of the error rates, the central marks indicate the median, the whiskers extend to the most extreme data points not considered outliers, and the outliers are plotted individually using the dot symbol. **c** The proportional number of available cameras by body part. The average proportions are: no cameras, 0.004%; 1 camera, 0.015%; 2 cameras, 0.038%; 3 cameras, 1.048%; 4 cameras, 98.895%. Abbreviation: n_cam. number of cameras, NaN. Not a Number.

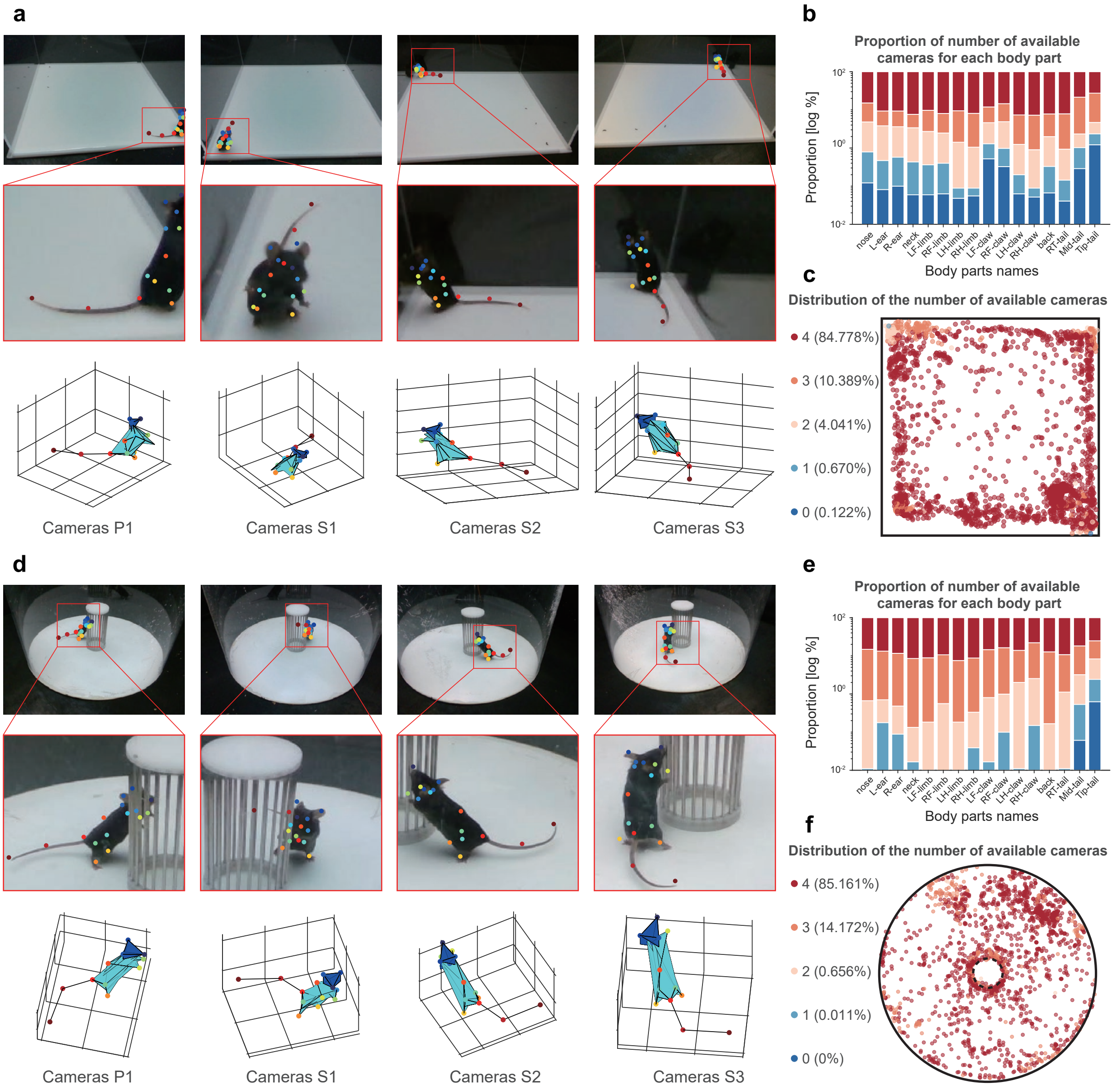

Supplementary Fig. 6 | Evaluation of the 3D reconstruction in cases of view-point–specific disappearances of body parts. a, **d** 2D pose tracking and 3D skeleton reconstruction of representative view-point disappearance frames from two different test apparatuses. **a** First test: square open field test. The behavior chamber is out of the field of view, and blind areas may occur when the animal enters the four corners; **d** Second test: circular open-field with a sociability cage. The mouse can easily be occluded by the cage, thus blind areas may exist in one or more perspectives. Top: selected frames with one or more views in which body parts disappear. Middle: magnification to show the disappearance details. Bottom: successfully reconstructed 3D skeletons shown in approximately the same views as the corresponding recordings. **b**, **e** The proportional number of cameras available for 3D reconstruction for each body part. In the first test, an average of 99.398 ± 0.149% of all frames showing the body part can meet the reconstruction requirements; In the second test, the average reconstruction rate of all body parts is 99.776 ± 0.150%. **c**, **f** The distribution of the number of available cameras for 3D reconstruction.

The color-coded dots indicate how many cameras are used for reconstruction at the indicated location. The positions of all the points are the x and y coordinates of the nose. For visualization purposes, the data are down-sampled to 10%. The proportional number of cameras used to reconstruct the nose in 3D is indicated in the key on the left.

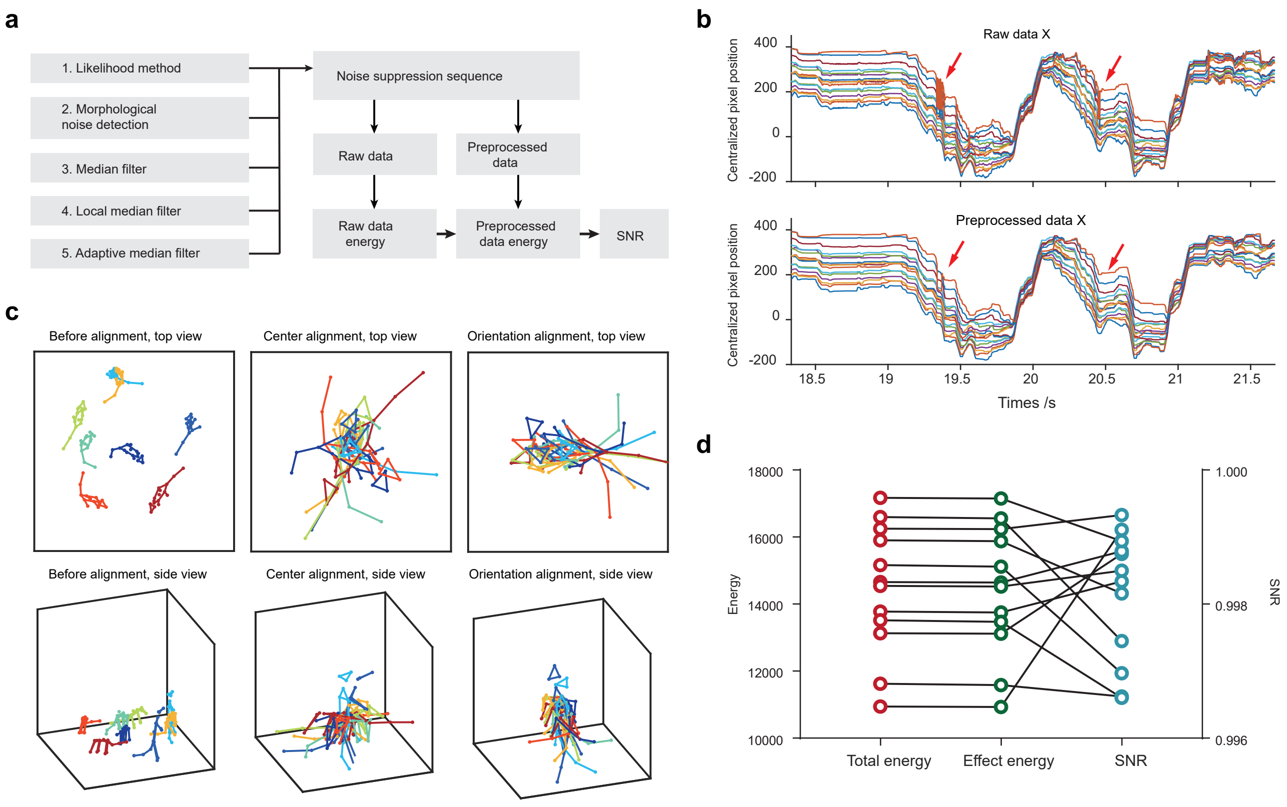

Supplementary Fig. 7 | The procedure of data preprocessing. a The procedure of data quality control contains two main parts, a noise suppression sequence part ordered by five filtering modules and the SNR detection part. **b** The selected multi-dimensional time series of mouse skeleton are preprocessed by a data quality control procedure. The red arrows indicate that the apparent noises are suppressed, and the whole series doesn't miss too much rapidly changing details. **c** Eight skeleton frames are randomly selected for a demo of mice alignment. The results of center orientation alignment are demonstrated on the top view and side view. **d** The multi-dimensional time-series data quality of 12 involved test mice are described, whose SNRs are more than 0.996, representing that there are averaged only four noise points in a thousand data points needed to be eliminated. Abbreviation: SNR. Signal-to-noise ratio.

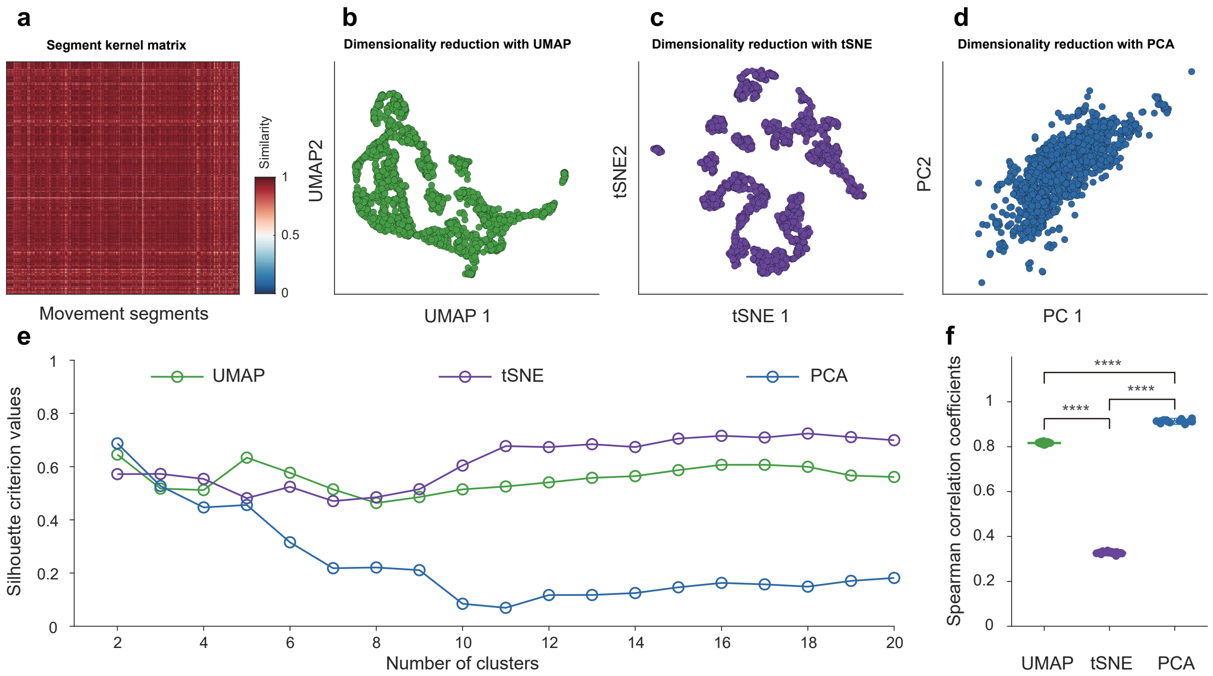

Supplementary Fig. 8 | Comparison of three algorithms of dimensionality reduction for the representation of the NM feature space structure. a Segment kernel matrix of a representative single-session behavioral experiment recording. The matrix pixels represent the normalized similarity value of 937 pairs of decomposed movement segments. **b-d** Dimensionality reduction with the three most-used algorithms: UMAP, tSNE (t-distributed stochastic neighbor embedding), and PCA (principal component analysis). For visualization purposes, the segment kernel matrix is reduced to two dimensions. **e** Quantification of local structure preservation by evaluation of the silhouette criterion values of the dimensionality reduction result of each algorithm. The silhouette criterion values are calculated by enumerating the clusters from two to twenty. The average silhouette criterion values are: UMAP, 0.557 ± 0.011; tSNE, 0.619 ± 0.021; PCA, 0.240 ± 0.039. **f** Quantification of global structure preservation by evaluation of the Spearman correlation coefficients between the original segment kernel matrix and the dimensionality-reducted result of each algorithm. For each algorithm, we first randomly subsampled 70% of the kernel matrix 20 times. Each time, the Spearman correlation coefficients are calculated between the selected segment kernel sub-matrix and the paired-wise distances of the dimensionality-reduced data. The average coefficients are: UMAP, 0.817 ± 0.001; tSNE, 0.326 ± 0.001; PCA, 0.913 ± 0.002. Statistics: ****UMAP V.S. tSNE, $P < 0.0001$, $q = 309.3$, $DF = 57$; ****UMAP V.S. PCA, $P < 0.0001$, $q = 1568$, $DF = 57$; ****tSNE V.S. PCA, $P < 0.0001$, $q = 466.1$, $DF = 57$ by one-way ANOVA followed by Tukey's multiple comparisons test. In box plots, the lower and upper edges of the box are the 25th and 75th percentiles of the values, the central marks indicate the median, the lower and upper whiskers are the minima, maxima values.

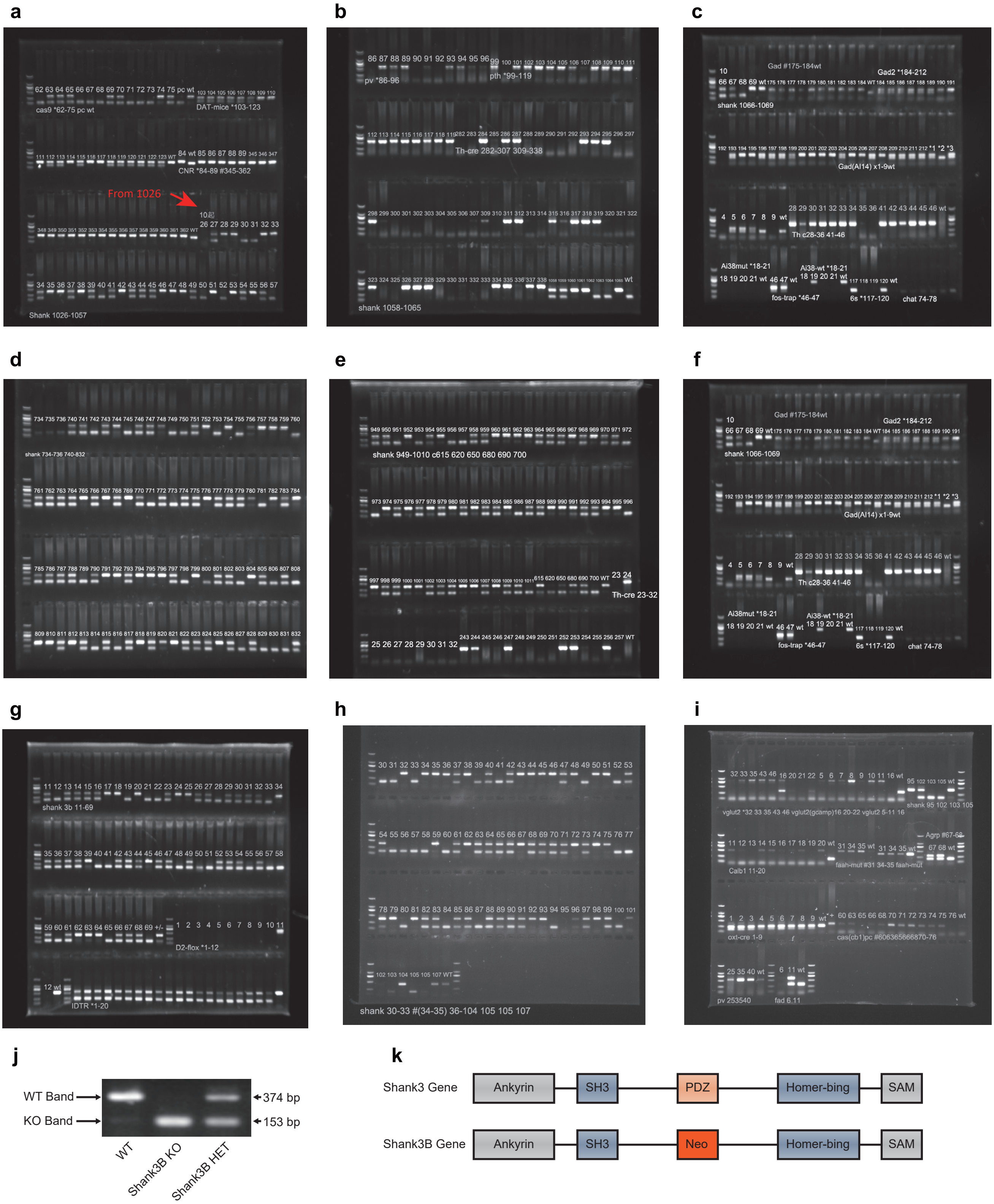

Supplementary Fig. 9 | Genotyping for Shank3 mice. a-h Full scans of PCR genotyping for the mice used in behavioral experiments. **i** Retested result for 95th sample due to unclear result in the first test. **j** PCR genotyping showing the *Shank3B*$^{+/+}$(WT), *Shank3B*$^{-/-}$ (*Shank3B* KO), and *Shank3B*$^{+/-}$ (*Shank3B* HET) mice. **k** Working strategy for *Shank3B* mutant mice. Abbreviation: SH3. Src-homology domain 3; PDZ. Postsynaptic density 95, PSD-85; Discs large, Dlg; Zonula occludens-1, ZO-1; Neo. neo cassette; SAM. The sterile alpha motif domain.

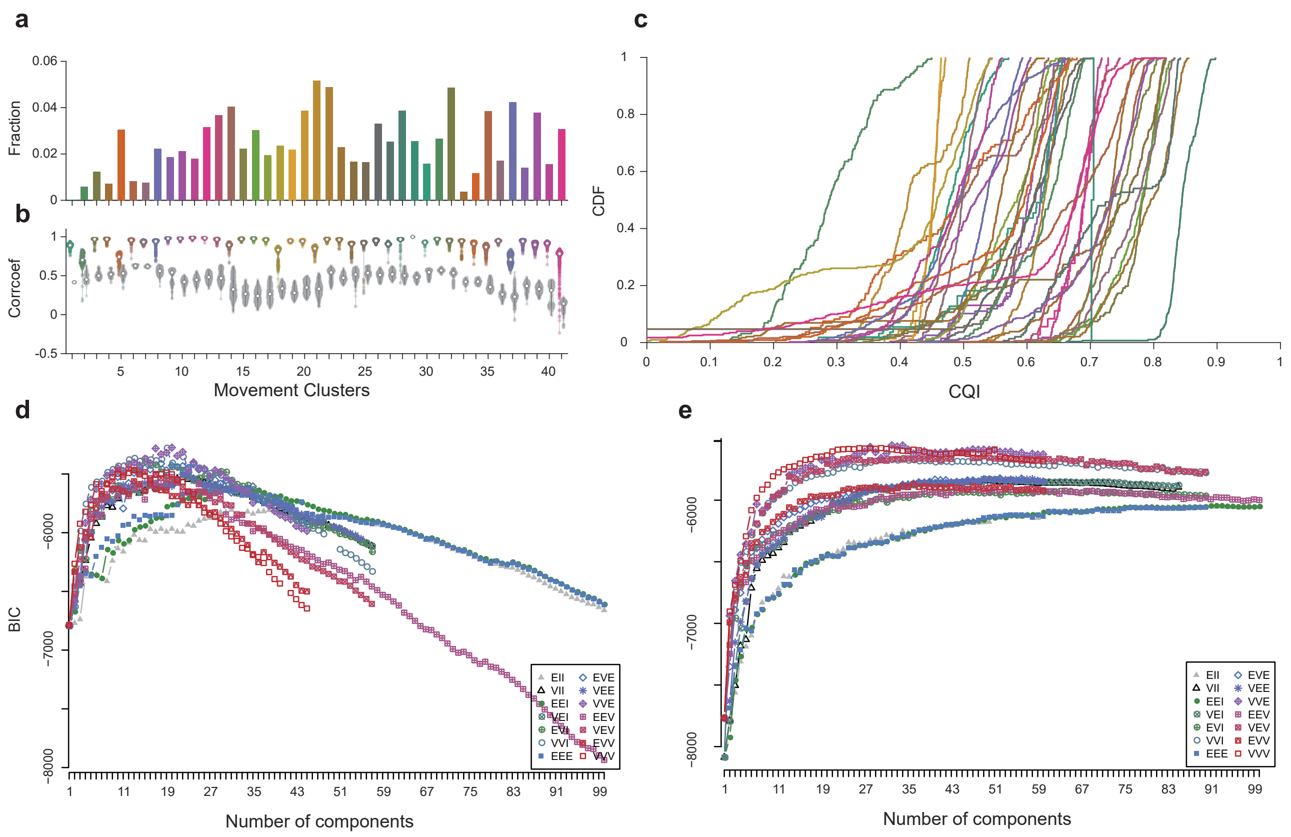

Supplementary Fig. 10 | Clustering quality evaluation and the number of clusters determination. **a** the fractions of movement bouts number (total number: 16607), the color-coded bars indicate the clustered movement types (totally 41 types). **b** the intra-CC (color-coded), and inter-CC (grey dots) of each movement group. The dots on each violin plot represents their intra-CC or inter-CC, and dots number in a pair of violin plot in each group are the same. **c** the cumulative distribution function of CQI of the movement clusters. The clusters represented by the curves on the right side have better clustering qualities, and their corresponding movements are more stereotyped. **d** the BIC of single-session experiment shows (related to Fig. 4) the most appropriate number of clusters in movement clustering could be chosen in the range of 10 to 20. **e** the BIC of all the mice's movements (related to Fig. 6) in movement space shows when the number of clusters is beyond about 20, the BIC of all the movements is tending to be steady, and the maximum BIC is in the range of 35 to 45. Abbreviation: CC. Correlation Coefficient, CDF. Cumulative Distribution Function, CQI. Clustering Quality Index, BIC. Bayesian Information Criterion.

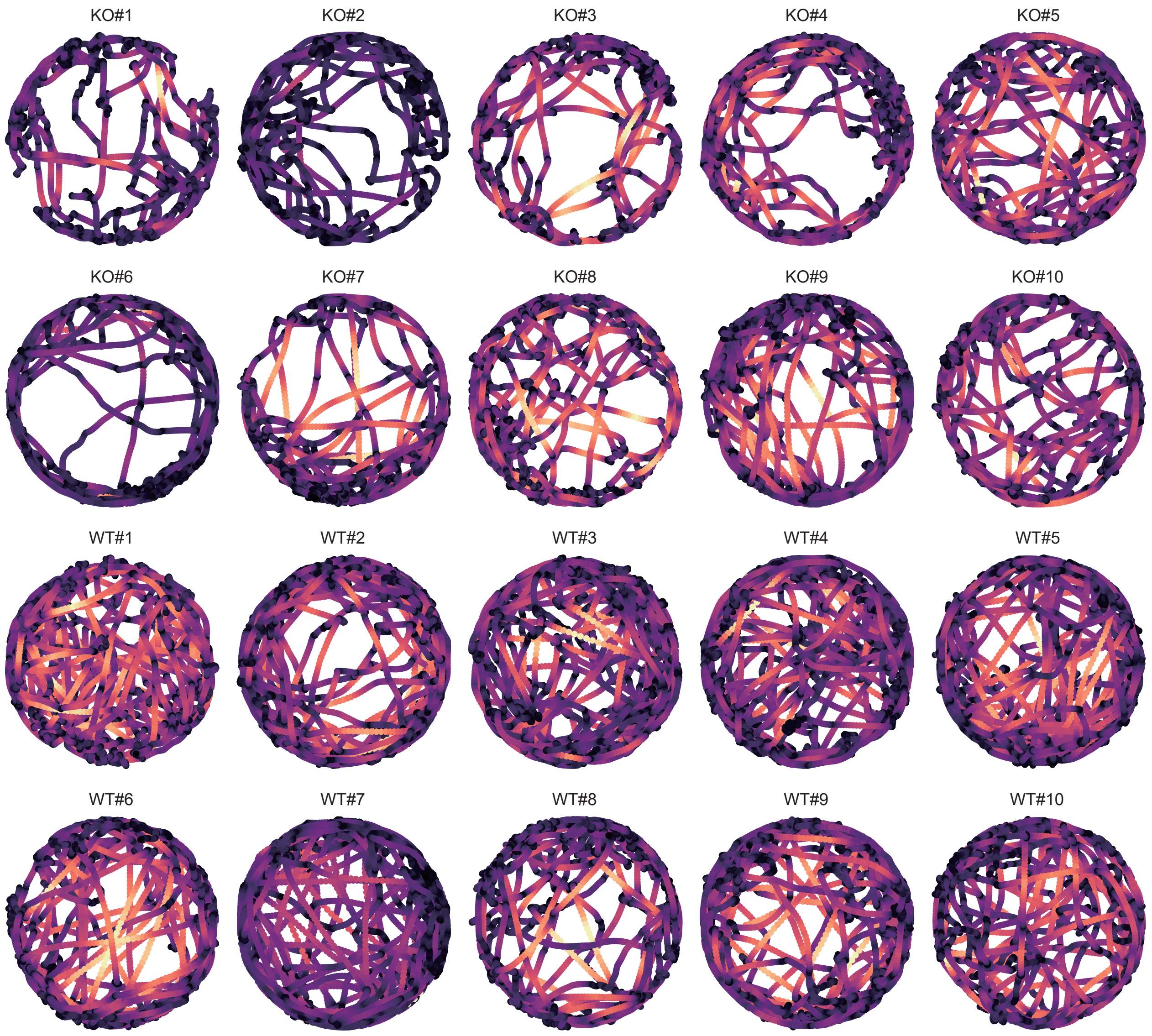

Supplementary Fig. 11 | The velocity-trajectory heatmaps of all the 10 *Shank3B*$^{-/-}$ (KO) mice and 10 *Shank3B*$^{+/+}$ (WT) mice. Compare the velocity-trajectory heatmaps between the two groups, KO mice have lower activity levels than WT mice.

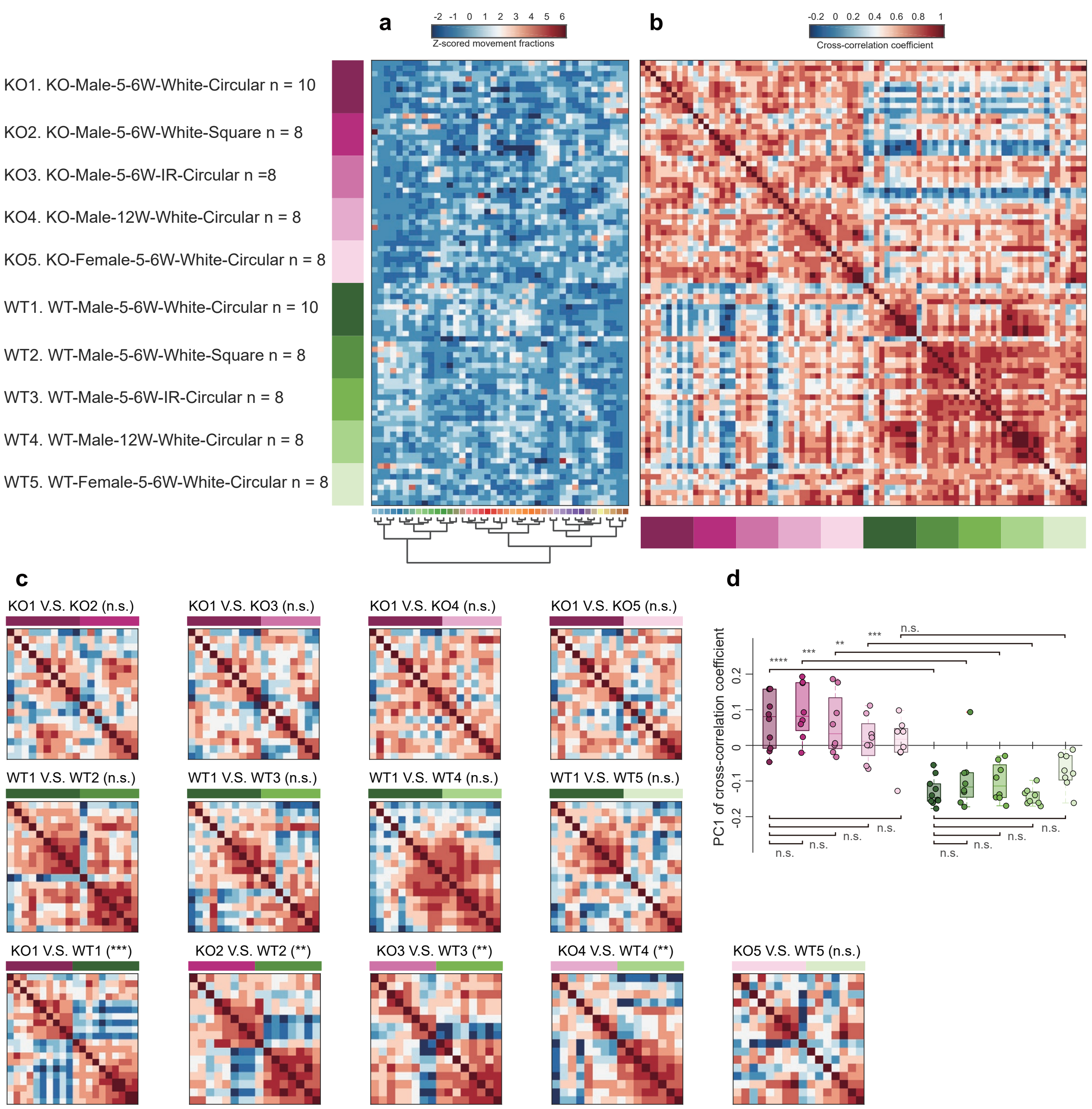

Supplementary Fig. 12 | Group comparison of Shank3B KO mice under different conditions. a The movement fraction matrix of mice in ten different groups. The color-bars shown in the left indicate the group conditions for the corresponding rows of the matrix (see Supplementary Methods for further details). Each row in the fraction matrix represents the tested mouse, and each column corresponds to the behavioral module types arranged with the dendrogram in the bottom. For visualization and comparison purpose, the values of movement fraction matrix are normalized with z-score by rows. In each group, the row orders are determined by placing the sample with largest variance of the movement fraction, and then the other samples are ranked according to the decreasing correlation with the first row. **b** The cross-correlation coefficients matrix (CCCM) of the movement fractions among all ten groups samples. **c** The group comparisons of behavioral correlations between the selected conditions,

which are shown with twelve submatrices of **b**. **d** The behavioral statistics between ten groups. The comparison metric is determined by calculating the principal component (PC) of the CCCM, then using the first PC (PC1) to evaluate the overall behavioral differences across ten groups (One-sided Kruskal-Wallis test followed by Dunn's multiple comparisons test, ****KO1 V.S. WT1, $P < 0.0001$; ***KO2 V.S. WT2, $P < 0.0006$; **KO3 V.S. WT3, $P = 0.002$, *** KO4 V.S. WT4, $P = 0.0005$). In box plots, the lower and upper edges of the box are the 25th and 75th percentiles of the values, the central marks indicate the median, the lower and upper whiskers are the minima, maxima values.

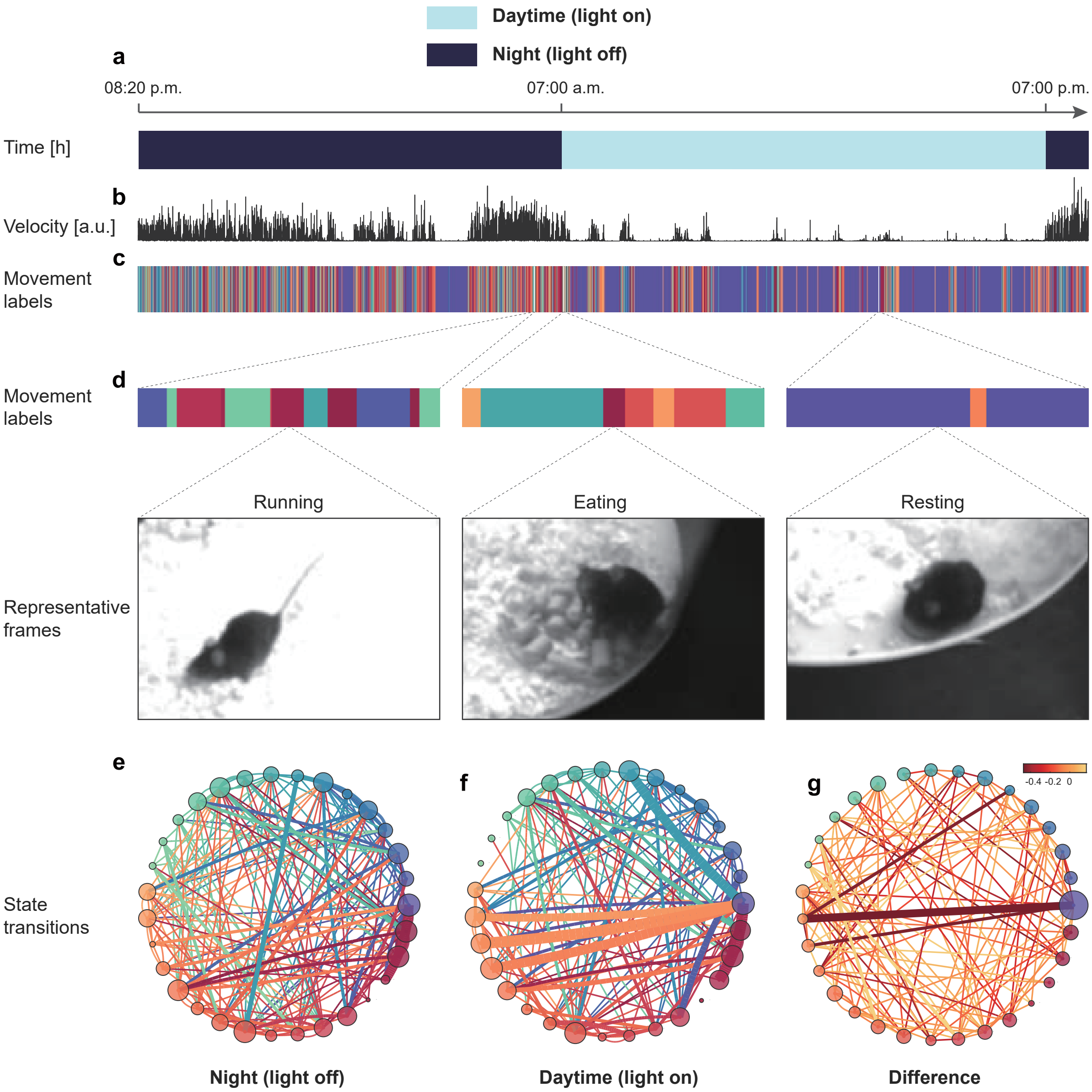

Supplementary Fig. 13 | Continuous long-term monitoring and analysis of mouse behavior. a The timeline of the behavioral recording period over 24 hours. **b** The normalized velocity of the mouse across 24 hours aligned to the timeline. **c** The decomposed behavioral modules shown with color-coded labels. **d** Three magnified representative behavioral modules and selected, single corresponding frames. Left, running on the litter; Middle, eating; Right, prolonged immobility resembling resting. **e, f** State transitions of the movement modules in night and day phases. **g** Differences in the state transitions between night and day. The color of the dots in **e, f,** and **g** correspond to the behavioral modules shown in **c**. The size of the dots represents the rank of the module probabilities over 24 hours. The color of the connections in **e** and **f** represents the direction from the previous state to the current state, and its color is the same as that of the previous state. The width of the connections in **e** and **f** represents the normalized two-state transition probability. The color and width of the connections in **g** represent the normalized difference between **e** and **f**.

Supplementary Notes

Calibration of the 3D motion capture system

We used MATLAB's StereoCameraCalibrator GUI to calibrate the cameras' 3D motion capture system. The GUI uses the basic principles of Zhang's calibration method [1]. Furthermore, we prepared checkerboard pictures to obtain calibration parameters.

First, we used the custom written Python code based on OpenCV to control the four cameras to capture checkerboard pictures synchronously. We used two different checkerboard display schemes in all our behavioral experiments. The old version uses a 10-inch tablet to display the checkerboard. The experimenter needs to control the synchronous image capturing of the four cameras by a keyboard shortcut, then move the checkerboard to different positions until each camera captures 70 images of the checkerboard (Supplementary Fig. 3a-d). To improve the efficiency of the checkerboard capturing step, we updated the 3D motion capture system. Thus, the new version uses a 32-inch displayer to show the checkerboards. Compared with the old version, the new version integrates the code of the checkerboard display, checkerboard moving, and four-camera checkerboard capturing, by using the 3D motion capture system to automatically capture the checkerboard images, thus not requiring manual operation (see Supplementary Fig. 2).

After capturing the checkerboard images, we estimated the camera parameters using the following equation, assuming that X, Y, and Z were the 3D coordinate points of the stereo cameras:

$$P\begin{pmatrix} u \\ v \\ 1 \end{pmatrix} = \begin{pmatrix} \alpha & \gamma & u_0 \\ 0 & \beta & v_0 \\ 0 & 0 & 1 \end{pmatrix} \begin{pmatrix} \mathbf{R} & \mathbf{t} \end{pmatrix} \begin{pmatrix} X \\ Y \\ Z \\ 1 \end{pmatrix} \quad (1),$$

where P is a scaling constant, u and v are the pixel coordinates of the checkerboard image, α is the focal length divided by the length of the picture, β is the focal length divided by the width of the picture, γ is the radial distortion parameter, u_0 and v_0 are midpoints in pixel coordinates of the image, $\mathbf{R}$ is a rotation matrix, and $\mathbf{t}$ is the translation vector. Zhang's calibration method can obtain , β, γ, $\mathbf{R}$, $\mathbf{t}$, and P. Each camera acquired 70 calibration photos, summing to a total of 280 calibration photos by the four cameras (Supplementary Fig. 3). Moreover, we measured and recorded the grid's checkerboard size. We set one of the cameras as the primary camera and the remaining three cameras as sub-cameras. The cameras were divided into three groups, each containing a secondary camera and the primary camera (see Supplementary Fig. 4). In each group, we used StereoCameraCalibrator GUI to calibrate the two cameras and obtain three calibrated files.

Mouse pose estimation

The DeepLabCut (DLC) toolbox[2] was used to track the animal's 2D body features from the four separately recorded videos. We manually labeled about 3000 images as a training set to improve the environment adaptation of the DLC pose estimation model. The maximum number of iterations was 1,200,000, and the final cross-entropy loss was 0.017. The training step took 20 hours on the NVIDIA GTX 2080Ti GPU. We used the trained network model to track a new video and obtain 16 feature points at each video frame, including the nose, neck, left front limb, right front limb, left hind limb, right hind limb, left front claw, right front claw, left hind claw, right hind claw, back, root tail, middle tail, and tip tail. Each video produced one feature file, and we obtained four feature files per experimental trial.

3D pose reconstruction

We used pose3d toolbox in MATLAB to perform the 3D reconstruction of the animal skeleton. This toolbox uses the triangulation algorithm to obtain 3D data, according to the following equation:

$$\mathbf{x}_i = \mathbf{P}_i\mathbf{X} \tag{2},$$

where $\mathbf{x}_i = [m_i, n_i, 1]^T$, $\mathbf{X}=[I, J, K, 1]^T$, and $\mathbf{P}_i = \mathbf{A}_i[\mathbf{R}_i, \mathbf{t}_i]$. $\mathbf{x}_i$ is the coordinate of the matching point in the i camera image; $\mathbf{X}$ is a vector of 3D point; I , J , K are the 3D points of $\mathbf{X}$; $\mathbf{P}_i$ is the projection matrix; $\mathbf{A}_i$ is the internal reference of the ith camera; $\mathbf{R}_i$ is the rotation matrix of the ith camera; and $\mathbf{t}_i$ is the ith translation vector of the ith camera. In order to obtain $\mathbf{X}$, we used the solution of the least square method, obtained by singular value decomposition [3]. Specifically, three calibrated files and four feature files were used as input to the system of pose3d, and the system produced one 3D feature file as output. After this step, we rotated and translated the 3D coordinates to a horizontal direction for visualization and subsequent processing. To show the 3D reconstruction effect, we used the Delaunay triangulation [4] to fill the skeleton.

Data pre-processing

Data quality control

Data quality control (Supplementary Fig. 7a) was performed to ensure the precision of follow-up algorithms. It included two steps: data noise suppression and data quality assessment.

Data noise suppression: We built a suppression algorithm sequence of data noise, including a likelihood method, morphological noise detection, median filter [5], local median filter, and adaptive median filter. These algorithms were ordered as needed. The likelihood method involved a noise detection step. The likelihood of raw data in DLC was assessed, and a threshold was set to distinguish the noise and non-noise level likelihoods. We set the threshold to 95%; when the likelihood was smaller than 95%, the noise data points were detected. Morphological noise detection was used to identify a leap of body points at the physical morphological level. The most precise pose estimation frame was

selected automatically by the maximum likelihood frame as a template. The maximum body points distance of the template multiplied by a coefficient was set as a threshold to detect morphological saltation of animal skeletons by comparing the current maximum body points distance with the threshold. Next, the distance between two points, higher than the threshold, was marked as noise. The median filter is a widely used method for impulse noise. It replaces the impulse noise points by median values in a time window, which can effectively eliminate the impulse noise while keeping the high-frequency details of data. Most noise data points in pose estimation can be seen as impulse noise; thus, the median filter was suitable to suppress them. The local median filter was used as an interpolation algorithm in our framework. We placed the median filter window across the noise data points detected by the likelihood method and morphological filter, and then replaced the noise data points by median data in the filter window. Since this filter only focuses on noise location, it does not influence the qualified data. The adaptive median filter is designed to substitute the median filter. The median filter has a fixed window width, thus treating all data at the same time scale. The adaptive median filter adopts a variable window width; thus, the impulse noise in different time scales can be disposed of more properly. We provide a default parameter of noise suppression algorithms sequence for preprocessing our 3D animal pose estimation data (likelihood method, morphological noise detection, and median filter with 1-s time window).

Data quality assessment (Supplementary Fig. 7d): We calculated the multi-dimensional time series total energy of raw data, preprocessed data, and noise defined as the absolute value of the subtraction of raw data and preprocessed data. Next, we used these energies to assign a signal-to-noise ratio (SNR). The division of preprocessed data was used to calculate the SNR's total energy to the raw data total energy.

Mouse alignment

Mouse alignment included four steps (Supplementary Fig. 7c), and all alignments were carried out in a 3D Cartesian coordinate system: $\mathbf{\Theta} = \{\mathbf{X}, \mathbf{Y}, \mathbf{Z}\}$.

Step 1-Center alignment: We considered the back point as a center point and subtracted this value from the value of each body part in plane $\{\mathbf{X}, \mathbf{Y}\}$, so that each frame of mouse skeleton would have the same center coordinate value (0, 0) after center alignment.

Step 2-Orientation alignment: We considered the vector from the back point to the tail root point as the orientation vector of each mouse. Thus, aligning the orientation of mice was similar to aligning the orientation of the orientation vector. We then computed rotation matrices from the orientation vector; the positive direction of the $\mathbf{X}$ axis of each frame rotated the mouse skeleton to the same orientation by the rotation matrices in plane $\{\mathbf{X}, \mathbf{Y}\}$. The data points in $\mathbf{Z}$ were not changed, thereby retaining the height information of the mouse skeleton.

Step 3-Size normalization: We determined that the size of mice could introduce bias in the decomposition of poses and movements because the weight of the body size is always larger than that of body movement in different mice. Considering that the length of the orientation vector could represent the size of the mouse skeleton, we calculated the distribution of orientation vector lengths of each mouse and chose the median value as the length pattern. Next, we set a standard length (practical value based on our tests on the range of the orientation vector acquired by our apparatus, which was set to 25) and calculated a list of size normalization factors by dividing the median value of orientation vector length with the standard length. All frames of skeletons were multiplied by the factors in different mice to correct the mouse size to a standard size.

Following the mice alignment procedure, there were some dimensions of time series that could be reduced. The center point series in plane $\{\mathbf{X}, \mathbf{Y}\}$ and the endpoint of orientation vectors in the Y axis were all zeros and would therefore only influence the time consumption but not the decomposition precision. Moreover, we focused on the body movements of mice. Previous reports have suggested that tail movements are relatively independent from those of the rest of the body[6]. Considering that rodents also emit other specific signals corresponding to other body parts (with the exception of the torso) through tail movements, the following motion quantification did not involve the motion features of the two parts of the tail. As our framework measured the inherent dynamic similarity of the time-series, the choice of features depended on the behavior of interest. Therefore, we removed nine dimensions from the 48 dimensions of the mouse time series and used 39 dimensions for the decomposition of poses and movements.

Behavioral decomposition

Behavioral decomposition was applied to non-locomotor movements (NM). First, we set $\mathbf{X} = \{\mathbf{x}_1, \mathbf{x}_2, ..., \mathbf{x}_{39}\}^T$ as the 39-dimension (not including 3 points of the tail) time series after alignment, where each time series $\mathbf{x}_i = (x_i^1, x_i^2, ..., x_i^N)^T$ contains $1 \times N$ ordered real values. $\mathbf{X}$ represented the reduced spatial dimensions, from 39 to 2 dimensions, by uniform manifold approximation and projection (UMAP) [7], which could keep the global and local structures of data at the same time. We then set $\mathbf{Z} = \{\mathbf{z}_1, \mathbf{z}_2\}^T$ as the dimension reducing time series and f_{UMAP} as the mapping of $\mathbf{X}$ to $\mathbf{Z}$ to obtain the following equation:

$$\mathbf{Z} = f_{UMAP}(\mathbf{X}) \tag{3},$$

where $f_{UMAP}(\cdot)$ includes the parameters n_neighbors, set to 80, and min_dist, set to 0.3.

Second, we used temporal reduction to decompose poses from $\mathbf{Z}$. The temporal reduction involved density clustering in the time dimension to cluster $\mathbf{Z}$ into k_{poses} clusters. The time dimension was represented by the distribution in which different centers of density can be seen as different poses

in $\mathbf{A}$. We used default parameters of the density clustering method in UMAP to obtain the results of pose decomposition. We set $\mathbf{U}=\{\mathbf{u}_1,\mathbf{u}_2\}^T$ as the poses decomposition time series, $\mathbf{l}=\{l_1,l_2,..,l_N\}^T$ as the clusters label, and f_{DC} as the density clustering mapping of $\mathbf{Z}$ to $\mathbf{U}$, to obtain the following equation:

$$\mathbf{U}=f_{DC}(\mathbf{Z}) \tag{4},$$

where $\mathbf{U}\in \mathrm{IR}^{2\times M}$, with M being the number of poses, calculated by the temporal reduction length r_L and continuous pose frames $\mathbf{Z}_{[p,p+L_p]}$. In the latter, $p\in[1,M]$, and L is the number of continuous pose frames. Each $\mathbf{Z}_{[p,p+L_p]}$ was decomposed into $\mathbf{Z}_{[p,p+l_p^j]}$ by r_L, and l could be calculated as follows:

$$l_p^j=\begin{cases} r_L, j<\mathrm{ceil}(\frac{L_p}{r_L}) \\ \mathrm{rem}(L_p,r_L), j=\mathrm{ceil}(\frac{L_p}{r_L}) \end{cases} \tag{5},$$

where $\mathrm{ceil}(\cdot)$ is rounded up to an integer and $\mathrm{rem}(\cdot)$ is the remainder. Next, we obtained a mapping list of $\mathbf{Z}_{[p,p+l_p^j]}\rightarrow\mathbf{U}_p$. The r_L was set to 5.

Third, we calculated the distance kernel matrix as $\mathbf{K}=\phi(\mathbf{U})^T\phi(\mathbf{U})\in \mathrm{IR}^{m\times m}$, where $\mathbf{K}=\{\mathbf{k}_1,\mathbf{k}_2,...,\mathbf{k}_M\}^T$ and $\mathbf{k}_i=(\kappa_i^1,\kappa_i^2,...,\kappa_i^M)^T$ were calculated from $\mathbf{U}$. κ_i^j defines the similarity between $\mathbf{U}_i$ and $\mathbf{U}_j$, where we used the Gaussian kernel [8], as follows: $\kappa_i^j=\exp\left[-\frac{(\mathbf{U}_i-\mathbf{U}_j)(\mathbf{U}_i-\mathbf{U}_j)^T}{2\sigma^2}\right]$. We fixed σ to 30 to keep each $\mathbf{U}$ from different videos having the same Gaussian kernel and mapping to the same high-dimensional space.

Fourth, NMs were decomposed via aligned cluster analysis (ACA) [9,10] according to $\mathbf{K}$. ACA involved two steps: initialization and optimization. We used spectral clustering (SC) [11] for initialization. We set $\begin{bmatrix}\mathbf{D}_{11} & \cdots & \mathbf{D}_{1P}\\ \vdots & \ddots & \vdots \\ \mathbf{D}_{P1} & \cdots & \mathbf{D}_{PP}\end{bmatrix}$=$\mathbf{K}$ as the SC initialization distance kernel matrix, where each $\mathbf{D}=\{\mathbf{d}_1,\mathbf{d}_2,...,\mathbf{d}_p\}^T$ is the initial NM matrix and $\mathbf{d}=(d_i^1,d_i^2,...,d_i^p)^T$. We set the cluster number k_m of SC to 30. Then, dynamic time alignment kernel (DTAK) was used as a metric to optimize ACA. $\mathbf{C}$ was the cumulative kernel matrix of $\mathbf{D}$ and was defined as $\mathbf{C}=\{\mathbf{c}_1,\mathbf{c}_2,...,\mathbf{c}_p\}^T$, where $\mathbf{c}$ is defined as

$\mathbf{c}=(c_i^1,c_i^2,...,c_i^p)^T$. The DTAK uses $\mathbf{C}$ and dynamic programming to calculate the similarity of two time series as follows:

$$\tau(\mathbf{c}_i,\mathbf{c}_j)=\frac{\mathbf{C}_p}{p_x+p_y},c_i^j=\max\begin{cases}c_{i-1}^j+d_i^j\\c_{i-1}^{j-1}+2d_i^j\\c_i^{j-1}+d_i^j\end{cases} \tag{6}$$

Then, the energy function of ACA was written as follows:

$$J_{aca}(\mathbf{G},\mathbf{s})=\sum_{i=1}^{k_m}\sum_{j=1}^{P}g_{ij}\left\|\psi(\mathbf{V}^i_{[s_j,s_{j+1})})-\psi(\mathbf{z}_i)\right\|^2,\ \ n_{\min}\le s_{j+1}-s_j<n_{\max} \tag{7}$$

$$\left\|\psi(\mathbf{V}^i_{[s_j,s_{j+1})})-\psi(\mathbf{z}_i)\right\|^2=\tau_{jj}-\frac{2}{p_i}\sum_{q=1}^{p}g_{iq}\tau_{jq}+\frac{1}{p_i^2}\sum_{q_1,q_2=1}^{p}g_{iq_1}g_{iq_2}\tau_{q_1q_2} \tag{8),}$$

where $\mathbf{G}\in\{0,1\}^{k_m\times P}$ is a cluster indicator matrix, $\mathbf{s}$ is a vector containing the start and end of each NM, g_{ij} is the element of $\mathbf{G}$ if $\mathbf{V}$ belongs to cluster i (g_{ij}=1), otherwise, g_{ij}=0 ; $\psi(\cdot)$ is the mapping of the sequence into a feature space; $\mathbf{V}$ is an NM series optimized until the optimal time duration; and $\mathbf{z}$ is the center NM series of cluster i. $\left\|\psi(\mathbf{V}^i_{[s_j,s_{j+1})})-\psi(\mathbf{z}_i)\right\|^2$ represents the distance between two NM time series $\mathbf{V}$ and $\mathbf{z}$, which is calculated as (6), where τ is the DTAK of $\mathbf{V}$ and $\mathbf{z}$. In equation (5), $[s_j,s_{j+1}]$ requires a specific range, which means that the temporal scale of each NM is restricted. We set $n_{\min}$ to 100 ms and $n_{\max}$ to 2000 ms as the temporal scale range of NMs. After determining all the parameters, the J_{aca} could be optimized as follows:

$$\mathbf{G},\mathbf{s}=\underset{\mathbf{G},\mathbf{s}}{\arg\min}\,J_{aca}(\mathbf{G},\mathbf{s})=\underset{\mathbf{G},\mathbf{s}}{\arg\min}\sum_{i=1}^{k_m}\sum_{j=1}^{P}g_{ij}\left\|\psi(\mathbf{V}^i_{[s_j,s_{j+1})})-\psi(\mathbf{z}_i)\right\|^2 \tag{9),}$$

which can be converted to a recursive form:

$$J(\theta)=\min_{1<i\le\theta}\left(J(i-1)+\min_{\mathbf{G},\mathbf{s}}J_{aca}(\mathbf{G},\mathbf{s})\Big|_{\mathbf{V}_{[i,\theta]}}\right) \tag{10}$$

To optimize $\mathbf{G}$ and $\mathbf{s}$ faster, the DPSearch tool was used to reduce the computational cost of ACA (introduced in detail in [9,10]).

Group segment kernel matrix and low dimensional embedding

Following the optimization of ACA, the optimal $\mathbf{G}$ and $\mathbf{s}$ were confirmed. We set $\mathbf{X}=\{\mathbf{x}_1,\mathbf{x}_2,...,\mathbf{x}_{39}\}^T$ as the NM time series and $\mathbf{x}_i=(x_i^1,x_i^2,...,x_i^N)^T$ as the data points of NMs after

alignment, spatial reduction, and temporal reduction. The optimal DTAK (Supplementary Fig. 7) distance matrix $\mathbf{T}$ could be calculated as follows:

$$\mathbf{T} = \begin{pmatrix} tdist(\mathbf{X}_{s_1}, \mathbf{X}_{s_1}) & \cdots & tdist(\mathbf{X}_{s_1}, \mathbf{X}_{s_P}) \\ \vdots & \ddots & \vdots \\ tdist(\mathbf{X}_{s_P}, \mathbf{X}_{s_1}) & \cdots & tdist(\mathbf{X}_{s_P}, \mathbf{X}_{s_P}) \end{pmatrix} \quad (11),$$

where $tdist(\mathbf{X}_{s_i}, \mathbf{X}_{s_j}) = \left\| \psi(\mathbf{X}_{s_i}) - \psi(\mathbf{X}_{s_j}) \right\|^2$ is the DTAK distance between two NMs dynamic $\mathbf{X}_{s_i}$ and $\mathbf{X}_{s_j}$.

Next, $\mathbf{T}$ was reduced from P dimensions to 2 dimensions by UMAP, which enabled large amounts of data to be more widely distributed. We set $\mathbf{Y} = \{\mathbf{y}_1, \mathbf{y}_2\}^T$ as the two-dimension UMAP embedding of NMs, where each dimension $\mathbf{y}_i = (y_i^1, y_i^2, ..., y_i^P)^T$ contains the position of NMs in embedding. Thereafter, the velocity of each NM segment was added to the embedding as the third dimension. The velocity dimension represented the locomotion of animals. We set $\mathbf{v} = (v_1, v_2, ..., v_P)^T$ as the velocity dimension and $\mathbf{C} = \{\mathbf{c}_1, \mathbf{c}_2, \mathbf{c}_3\}^T$ as the centroid of mice, to calculate the mean velocity of each segment as follows:

$$v_i = \frac{Fs}{m_i} \left\| \Delta \mathbf{C}_{s_i} \right\|^2 \quad (12),$$

where Fs=30 is the framerate of each video, m is the duration of the centroid segment $\mathbf{C}_s$, and $\Delta\mathbf{C}_s$ is the difference of $\mathbf{C}_s$. The velocity dimension was added as an orthogonal dimension to $\mathbf{Y}$; thus, we obtained 3D embedding as $\mathbf{E} = \{\mathbf{y}_1, \mathbf{y}_2, \mathbf{v}\}^T$. The 3D embedding represented the patterns of NM and locomotion, built as a whole mouse movement space. To compare the three dimensions in the same unit, we removed the unit using the Z-score in each dimension of $\mathbf{E}$. $\mathbf{E}_z = \{\mathbf{z}_1, \mathbf{z}_2, \mathbf{z}_3\}^T$ denoted the normalized embedding of $\mathbf{E}$ after Z-scoring [12].

Unsupervised clustering

Hierarchical clustering [13,14] was used to cluster 3D embedding. The pairwise distance matrix $\mathbf{D}$ between $\mathbf{E}_z$ was calculated using a standardized Euclidean distance [15]. Next, we used the inner squared distance [16] to obtain the linkage of $\mathbf{D}$. The number of clusters was set to 11 and 41 in single- and multi-videos embedding clustering, respectively, which was used to cut the hierarchical cluster tree. Hence, we obtained the 3D embedding label and the movement space for the further analysis of mouse movements.

Clustering quality index (CQI)

To quantify the quality of clustering, we defined CQI (Supplementary Fig. 10). Setting the DTAK distance matrix as $\mathbf{T}=[\mathbf{t}_1,\mathbf{t}_2,...,\mathbf{t}_N]^T$, the movements features of the same cluster were $\mathbf{T}_m^{N_m}=[\mathbf{t}_m^1,\mathbf{t}_m^2,...,\mathbf{t}_m^{N_m}]^T$, where N_m is the number of m movements. We first calculated the cross-correlation coefficient of intra and inter of clusters:

$$\begin{cases}\mathbf{c}_{\text{intra}}=xcorr(\mathbf{T}_m^{N_m^1},\mathbf{T}_m^{N_m^2})\\ \mathbf{c}_{\text{inter}}=xcorr(\mathbf{T}_m^{N_m^1},\mathbf{T}^{N_m^3})\end{cases} \tag{13}$$

where $\mathbf{c}_{\text{intra}}\in[-1,1]$ is the intra cross-correlation coefficient column vector of m movements, $\mathbf{c}_{\text{inter}}\in[-1,1]$ is the inter cross-correlation coefficient column vector of m movements, $\mathbf{T}_m^{N_m}$ is randomly divided into $\mathbf{T}_m^{N_m^1}$ and $\mathbf{T}_m^{N_m^2}$ in equal and $N_m^1+N_m^2=N_m$, $\mathbf{T}_m^{N_m^3}$ is randomly selected from a complementary set of $\mathbf{T}_m^{N_m}$, and $\mathbf{T}_m^{N_m^1}$ has the same number of $\mathbf{T}_m^{N_m^3}$. $\mathbf{c}_{\text{intra}}$ represents the similarity in a cluster of movements, and $\mathbf{c}_{\text{inter}}$ represents the similarity between a cluster of movements and other clusters of movements. Next, we used $\mathbf{c}_{\text{intra}}$ and $\mathbf{c}_{\text{inter}}$ to build a cartesian coordinate system, as follows: $\mathbf{\Theta}=\{\mathbf{c}_{\text{inter}},\mathbf{c}_{\text{intra}}\}$. The better the quality of clustering, the closer to 1 is the value of $\mathbf{c}_{\text{intra}}$, and closer to -1 is the value of $\mathbf{c}_{\text{inter}}$. Thus, we mapped $\mathbf{\Theta}$ to a new one-dimensional linear space, as follows:

$$\mathbf{\Phi}=\mathbf{A}\cdot[\mathbf{c}_{\text{intra}},\mathbf{c}_{\text{inter}}]^T \tag{14}$$

where $\mathbf{A}=[-\frac{\sqrt{2}}{2},\frac{\sqrt{2}}{2}]$ is the projection matrix from $\mathbf{\Theta}$ to $\mathbf{\Phi}$. In $\mathbf{\Theta}$, $\mathbf{\Phi}$ could be regarded as a linear function $c_{\text{intra}}+c_{\text{inter}}=0$, effectively representing the relationships between $\mathbf{c}_{\text{intra}}$ and $\mathbf{c}_{\text{inter}}$. Lastly, we normalized $\mathbf{\Phi}$ to $[0,1]$ by a sigmoid function to reduce the dynamic range of $\mathbf{\Phi}$ and calculated the CQI as follows: $\text{CQI}=\text{sigmoid}(\mathbf{\Phi})$.

Clustering quality correlation analysis

To compare the clustering similarity of inter and intra movement phenotype clusters simultaneously, we applied a correlation analysis with a linear regression [17]. The feature vector of each behavioral module ($1\times n$ vector) in the DTAK distance matrix ($n\times n$) represented the distance between each behavioral module, such that all dimensions of the feature vector would have the same unit to be compared. If two behavioral modules show a higher similarity, the correlation of their feature vector should also be similar. As such, we chose one behavioral module's feature vector as the reference and randomly selected the feature vector of other behavioral modules as the targets for comparison. Each target with the reference formed a $2\times n$ list, which was plotted on the two-dimensional space for

linear regression and correlation analysis. The higher the R value of the linear regression, the higher the correlation of the two behavioral modules.

For inter clustering, we sequentially chose the behavioral module as reference and randomly selected three other behavioral modules' feature vectors in the same cluster as targets to plot in the two-dimensional space until all references were plotted with targets. Next, we performed linear regression. For intra clustering, we sequentially chose the behavioral module as reference and randomly selected three other behavioral modules' feature vector out of the reference's cluster. We then plotted them in the two-dimensional space in gray points until all references were plotted with targets. Similarly, we performed linear regression. Notably, only the regression lines of the inter clusters are plotted in the figure 5b.

Mapping of new behavioral data to UMAP template

To compare the behavioral patterns of *Shank3B* mutant mice under different conditions, the 84 sets of behavior data were mapped into the same feature space. Processing such big data is often limited by computing resources. Therefore, we used the existing UMAP space that was constructed using the behavioral modules of 6 *Shank3B*$^{+/+}$ and 6 *Shank3B*$^{-/-}$ mice (data collected in an earlier experiment) as a template, and then used the UMAP toolbox in MATLAB and applied the default parameters to map the 84 behavioral datasets to the template [7,18]. Following the mapping, the categories of new behavioral modules were determined using K-Nearest-Neighbor to assign categories for new behavioral modules in low-dimensional embedding.

Moving intensity (MI)

To quantify the movement of each body part, we defined the MI. We considered each body part as a particle, which means that its moving intensity was correlated to velocity and accumulated over time. Further, the intensity of each movement segment was the summation across time, similar to the concept of energy. Therefore, we defined MI similar to kinetic energy in physics, which is the energy possessed by an object during its motion, as follows:

$$E_k = \frac{1}{2}mv^2 \tag{15}$$

where E_k is the kinetic energy, m is the mass of an object, and v is the velocity of an object. Considering that the body parts have unit mass and simplifying the calculation, we defined $m_p = \frac{1}{2}m$ as the mass of body parts, with the value of m_p being 1. Next, the kinetic energy was transformed to MI, and the MI of a movement in different coordinates was calculated as follows:

$$\begin{cases} E_{xy} = \frac{1}{2NT}\sum_{i=1}^{N}(\mathbf{V}_{x_i}^2 + \mathbf{V}_{y_i}^2) \\ E_{yz} = \frac{1}{2NT}\sum_{i=1}^{N}(\mathbf{V}_{y_i}^2 + \mathbf{V}_{z_i}^2) \\ E_{all} = \frac{1}{3NT}\sum_{i=1}^{N}(\mathbf{V}_{x_i}^2 + \mathbf{V}_{y_i}^2 + \mathbf{V}_{z_i}^2) \end{cases} \quad (16),$$

where E_{xy} is the mean MI in the XY coordinates plane, E_{yz} is the mean MI in the YZ coordinates plane, E_{all} is the mean MI in the XYZ coordinates space, N is the dimension of movements, $\mathbf{V}$ is the velocity matrix of different movements, and T is the temporal length of $\mathbf{V}$.

After each MI of a body part in XY (horizontal) and YZ (vertical) plane coordinates was calculated, it was visualized in Fig. 5. We visualized the MI in specific top (XY) and side views (YZ). First, we averaged the positions of each body part across time to plot the average pose skeleton of each movement category, as shown in solid lines. Second, we meshed the plane of the average pose skeleton to create grids to visualize the MI. Each position of the skeleton had a corresponding MI parameter which was plotted on the grids. The MIs in the same grids were averaged to obtain the mean MI. Third, we calculated the frequency of representation of each body part in a grid cell to calculate the weighted MI. This step aimed to assess the position-correlated MI of the body parts describable by the MI value. Finally, the weighted MIs were plotted as a heat map to observe the movement areas of each body part and depict the movement intensity in specific positions.

Traditional behavioral analysis

To quantify mouse behaviors in the circular open field test, we first plotted the velocity-trajectory maps for behavioral visualization and then calculated the mean velocity, maximum velocity, locomotion velocity [19], and mean anxiety index [20] of *Shank3B*$^{-/-}$ and *Shank3B*$^{+/+}$ mice (Supplementary Fig. 11). We extracted the back of each mouse in plane $\{\mathbf{X},\mathbf{Y}\}$ as the centroid of the mouse and applied a backward difference of centroids to calculate the instantaneous velocities of each frame. Next, the instantaneous velocities of each frame were normalized to $[0,1]$ and plotted at the positions of mouse centroids of each frame in the same canvas, corresponding to the velocity-trajectory map after drawing all the points.

The mean velocity V_m represented the mean activity of mice and was calculated as follows:

$$V_m = \frac{Fs}{N}\sum_{i=2}^{N}\sqrt{\left(x_c^i - x_c^{i-1}\right)^2 + \left(y_c^i - y_c^{i-1}\right)^2} \quad (17),$$

where $Fs=30$ is the framerate of each video, N is the number of frames in each video, and (x_c^i, x_y^i) is the mouse centroid of each frame in plane $\{\mathbf{X},\mathbf{Y}\}$. The maximum velocity $V_{\max}$ was calculated based on the instantaneous velocity $V_i = Fs\sqrt{\left(x_c^i - x_c^{i-1}\right)^2 + \left(y_c^i - y_c^{i-1}\right)^2}$, as follows:

$$V_{\max} = \max(\mathbf{V}) \tag{18}$$

where $\mathbf{V} = \left[V_2, V_3, ..., V_i\right]^T$ is the velocity vector.

The locomotion velocity V_l was calculated as follows:

$$V_l = \frac{1}{M}\sum_{i=2}^{M} V_i, V_i > V_{\mathbf{thres}} \tag{19}$$

where $V_{\mathbf{thres}}$ is the threshold of V_i to select the locomotion velocity from V_i set as 190, and M is the number of selected V_i.

The mean anxiety index represented the anxious degree of mice and was calculated as follows:

$$A_m = \frac{\sum\left[\mathbf{T}_m \wedge \left(\delta\left(x_{tc}, y_{tc}\right) * \mathbf{S}_{\frac{R}{2}}\right)\right]}{\sum \mathbf{T}_m} \tag{20}$$

where $\mathbf{T}_m$ is the trajectory map of each mouse, δ is the 2D impulse function with the same size of $\mathbf{T}_m$, (x_{tc}, y_{tc}) is the center of $\mathbf{T}_m$ that makes the impulse of δ locate to the center of $\mathbf{T}_m$, R is the radius of the circular open field, $\mathbf{S}_{\frac{R}{2}}$ is a disk structural element with the half radius of the circular open field, and $\delta\left(x_{tc}, y_{tc}\right) * \mathbf{S}_{\frac{R}{2}}$ is the 2D convolution of δ and $\mathbf{S}_{\frac{R}{2}}$ that sets the disk structural element at the center of $\mathbf{T}_m$. $\mathbf{T}_m \wedge \left(\delta\left(x_{tc}, y_{tc}\right) * \mathbf{S}_{\frac{R}{2}}\right)$ represents the extraction of the $\mathbf{T}_m$ of the central circular region in the range of $\mathbf{S}_{\frac{R}{2}}$ by AND operation, and $\sum(\cdot)$ sums over the values of the matrix elements.

State transitions analysis

For the behavioral analysis of continuous long-term monitoring, we applied a state transition analysis for the behavioral modules (Supplementary Fig. 13 e, f, g) [21–23]. The 31 behavioral modules were considered as states in the probabilistic graphical model. The probabilities of states were calculated approximately based on the fraction of the number of behavioral modules. The state transition

probabilities were calculated approximately based on the transition proportion of the number of previous behavioral modules to the current behavioral modules. The difference in the values of the transition probabilities between Night and Daytime were calculated approximately based on the difference between the behavioral modules' proportion of Daytime from Night-time.

Supplementary Tables

Supplementary Table 1 Definition of the behaviors (ethogram) for manual labeling

Behavior	Definition
Running	The mouse locomotes with relatively high speed.
Trotting	The mouse locomotes at a slow and intermittent pace.
Stepping	The mouse takes a step forward with a short distance locomotion.
Diving	The mouse bends down its body from standing state then stretches forward.
Sniffing	The mouse investigates environment with the nose held in the air or contacts the environment with nose closely.
Rising	The mouse rises from four legs on the ground to steadily stand on its hind legs.
Right turning	The mouse bends its body to right or turns body to right while walking.
Up stretching	The mouse stands on its hind legs while the front part of body stretches back and forth.
Falling	The mouse takes its croup as pivot and gets down with straight back from standing on its hind legs.
Left turning	The mouse bends its body to left or turns body to left while walking.
Walking	The mouse locomotes with relatively low speed.
Rearing	The mouse stands on its hind legs, and the back is straight.
Hunching	The mouse stands on its hind legs while the back is bent.
Self-grooming	The mouse licks its fur, grooms with the forepaws, or scratches with any limb.

Supplementary Table 2 The SHANK3 mice used in group comparison

Experiments index	Date	Start time	Mice index (genotyping result)	Genotypes	Ages (weeks)	Sexes	Environments	Light conditions
1	20201224	13:41	1030 (Fig. S9a)	Shank3B$^{-/-}$	5-6	Male	Circular open field	White light
2	20201224	15:33	1038 (Fig. S9a)	Shank3B$^{+/+}$	5-6	Male	Circular open field	White light
3	20201224	16:27	1042 (Fig. S9a)	Shank3B$^{+/+}$	5-6	Male	Circular open field	White light
4	20201224	17:20	1051 (Fig. S9a)	Shank3B$^{+/+}$	5-6	Male	Circular open field	White light
5	20201225	12:05	765 (Fig. S9d)	Shank3B$^{+/+}$	11-13	Male	Circular open field	White light
6	20201225	12:33	771 (Fig. S9d)	Shank3B$^{-/-}$	11-13	Male	Circular open field	White light
7	20201225	13:00	742 (Fig. S9d)	Shank3B$^{-/-}$	11-13	Male	Circular open field	White light
8	20201225	13:27	789 (Fig. S9d)	Shank3B$^{-/-}$	11-13	Male	Circular open field	White light
9	20201225	13:54	810 (Fig. S9d)	Shank3B$^{+/+}$	11-13	Male	Circular open field	White light
10	20201225	14:22	812 (Fig. S9d)	Shank3B$^{+/+}$	11-13	Male	Circular open field	White light
11	20201225	14:49	814 (Fig. S9d)	Shank3B$^{-/-}$	11-13	Male	Circular open field	White light
12	20201225	15:16	818 (Fig. S9d)	Shank3B$^{+/+}$	11-13	Male	Circular open field	White light
13	20201225	16:12	753 (Fig. S9d)	Shank3B$^{-/-}$	11-13	Male	Circular open field	White light
14	20201225	17:08	1053 (Fig. S9a)	Shank3B$^{+/+}$	5-6	Male	Circular open field	White light
15	20201226	12:25	754 (Fig. S9d)	Shank3B$^{+/+}$	11-13	Male	Circular open field	White light
16	20201226	13:49	829 (Fig. S9d)	Shank3B$^{-/-}$	11-13	Male	Circular open field	White light
17	20201226	14:17	985 (Fig. S9e)	Shank3B$^{+/+}$	11-13	Male	Circular open field	White light
18	20201226	14:46	986 (Fig. S9e)	Shank3B$^{-/-}$	11-13	Male	Circular open field	White light
19	20201226	15:13	982 (Fig. S9e)	Shank3B$^{+/+}$	11-13	Male	Circular open field	White light
20	20201226	15:41	767 (Fig. S9d)	Shank3B$^{+/+}$	11-13	Male	Circular open field	White light
21	20201226	16:36	773 (Fig. S9d)	Shank3B$^{-/-}$	11-13	Male	Circular open field	White light
22	20201227	12:08	1032 (Fig. S9a)	Shank3B$^{+/+}$	5-6	Female	Circular open field	White light
23	20201227	12:36	1036 (Fig. S9a)	Shank3B$^{+/+}$	5-6	Female	Circular open field	White light
24	20201227	13:32	1050 (Fig. S9a)	Shank3B$^{-/-}$	5-6	Female	Circular open field	White light
25	20201227	14:00	1068 (Fig. S9f)	Shank3B$^{-/-}$	5-6	Female	Circular open field	White light
26	20201227	14:26	1061 (Fig. S9b)	Shank3B$^{+/+}$	5-6	Female	Circular open field	White light
27	20201227	14:53	1062 (Fig. S9b)	Shank3B$^{+/+}$	5-6	Female	Circular open field	White light
28	20201227	15:21	1063 (Fig. S9b)	Shank3B$^{-/-}$	5-6	Female	Circular open field	White light
29	20201227	16:16	1065 (Fig. S9b)	Shank3B$^{+/+}$	5-6	Female	Circular open field	White light
30	20201228	11:57	1029 (Fig. S9a)	Shank3B$^{+/+}$	5-6	Male	Square open field	White light
31	20201228	12:26	1030 (Fig. S9a)	Shank3B$^{-/-}$	5-6	Male	Square open field	White light
32	20201228	12:53	1031 (Fig. S9a)	Shank3B$^{-/-}$	5-6	Male	Square open field	White light
33	20201228	13:21	1033 (Fig. S9a)	Shank3B$^{+/+}$	5-6	Male	Square open field	White light
34	20201228	13:49	1035 (Fig. S9a)	Shank3B$^{+/+}$	5-6	Male	Square open field	White light
35	20201228	14:18	1038 (Fig. S9a)	Shank3B$^{+/+}$	5-6	Male	Square open field	White light
36	20201228	14:46	1040 (Fig. S9a)	Shank3B$^{-/-}$	5-6	Male	Square open field	White light
37	20201228	15:14	1042 (Fig. S9a)	Shank3B$^{+/+}$	5-6	Male	Square open field	White light
38	20201228	15:41	1046 (Fig. S9a)	Shank3B$^{+/+}$	5-6	Male	Square open field	White light
39	20201228	16:08	1051 (Fig. S9a)	Shank3B$^{+/+}$	5-6	Male	Square open field	White light
40	20201228	17:02	1053 (Fig. S9a)	Shank3B$^{+/+}$	5-6	Male	Square open field	White light
41	20201229	14:04	1029 (Fig. S9a)	Shank3B$^{+/+}$	5-6	Male	Circular open field	Infrared
42	20201229	14:31	1030 (Fig. S9a)	Shank3B$^{-/-}$	5-6	Male	Circular open field	Infrared
43	20201229	14:59	1031 (Fig. S9a)	Shank3B$^{-/-}$	5-6	Male	Circular open field	Infrared
44	20201229	15:26	1033 (Fig. S9a)	Shank3B$^{+/+}$	5-6	Male	Circular open field	Infrared
45	20201229	15:54	1051 (Fig. S9a)	Shank3B$^{+/+}$	5-6	Male	Circular open field	Infrared
46	20201229	16:48	1053 (Fig. S9a)	Shank3B$^{+/+}$	5-6	Male	Circular open field	Infrared
47	20201229	17:15	1035 (Fig. S9a)	Shank3B$^{+/+}$	5-6	Male	Circular open field	Infrared

48	20201229	15:42	1038 (Fig. S9a)	Shank3B$^{+/+}$	5-6	Male	Circular open field	Infrared
49	20201229	18:10	1040 (Fig. S9a)	Shank3B$^{-/-}$	5-6	Male	Circular open field	Infrared
50	20201229	18:38	1042 (Fig. S9a)	Shank3B$^{+/+}$	5-6	Male	Circular open field	Infrared
51	20201229	19:03	1046 (Fig. S9a)	Shank3B$^{+/+}$	5-6	Male	Circular open field	Infrared
52	20201230	11:47	69 (Fig. S9g)	Shank3B$^{-/-}$	5-6	Male	Circular open field	White light
53	20201230	12:44	65 (Fig. S9g)	Shank3B$^{-/-}$	5-6	Male	Circular open field	White light
54	20201230	13:12	12 (Fig. S9g)	Shank3B$^{-/-}$	5-6	Male	Circular open field	White light
55	20201230	14:36	58 (Fig. S9g)	Shank3B$^{-/-}$	5-6	Male	Circular open field	White light
56	20201230	15:07	69 (Fig. S9g)	Shank3B$^{-/-}$	5-6	Male	Square open field	White light
57	20201231	11:43	65 (Fig. S9g)	Shank3B$^{-/-}$	5-6	Male	Circular open field	Infrared
58	20201231	12:11	12 (Fig. S9g)	Shank3B$^{-/-}$	5-6	Male	Circular open field	Infrared
59	20201231	13:04	57 (Fig. S9g)	Shank3B$^{-/-}$	5-6	Male	Circular open field	Infrared
60	20201231	13:32	58 (Fig. S9g)	Shank3B$^{-/-}$	5-6	Male	Circular open field	Infrared
61	20201231	14:47	12 (Fig. S9g)	Shank3B$^{-/-}$	5-6	Male	Square open field	White light
62	20201231	15:41	57 (Fig. S9g)	Shank3B$^{-/-}$	5-6	Male	Square open field	White light
63	20201231	16:09	58 (Fig. S9g)	Shank3B$^{-/-}$	5-6	Male	Square open field	White light
64	20201231	16:40	69 (Fig. S9g)	Shank3B$^{-/-}$	5-6	Male	Circular open field	Infrared
65	20210101	12:20	22 (Fig. S9g)	Shank3B$^{-/-}$	5-6	Female	Circular open field	White light
66	20210101	12:48	24 (Fig. S9g)	Shank3B$^{+/+}$	5-6	Female	Circular open field	White light
67	20210101	13:15	25 (Fig. S9g)	Shank3B$^{+/+}$	5-6	Female	Circular open field	White light
68	20210101	13:41	19 (Fig. S9g)	Shank3B$^{-/-}$	5-6	Female	Circular open field	White light
69	20210101	14:36	40 (Fig. S9g)	Shank3B$^{-/-}$	5-6	Female	Circular open field	White light
70	20210101	15:08	33 (Fig. S9g)	Shank3B$^{-/-}$	5-6	Female	Circular open field	White light
71	20210101	15:35	41 (Fig. S9g)	Shank3B$^{-/-}$	5-6	Female	Circular open field	White light
72	20210101	14:02	45 (Fig. S9g)	Shank3B$^{+/+}$	5-6	Female	Circular open field	White light
73	20210107	20:20	H1	C57BL/6J	13	Male	Circular home-cage	Infrared cameras (12 hours white light and 12 hours dark)
74	20200615	10:11	48 (Fig. S9h)	Shank3B$^{+/+}$	5-6	Male	Circular open field	White light
75	20200615	10:39	72 (Fig. S9h)	Shank3B$^{+/+}$	5-6	Male	Circular open field	White light
76	20200615	11:06	49 (Fig. S9h)	Shank3B$^{-/-}$	5-6	Male	Circular open field	White light
77	20200615	11:33	58 (Fig. S9h)	Shank3B$^{-/-}$	5-6	Male	Circular open field	White light
78	20200615	12:00	98 (Fig. S9h)	Shank3B$^{+/+}$	5-6	Male	Circular open field	White light
79	20200615	12:33	95 (Fig. S9i)	Shank3B$^{+/+}$	5-6	Male	Circular open field	White light
80	20200615	14:06	100 (Fig. S9h)	Shank3B$^{-/-}$	5-6	Male	Circular open field	White light
81	20200615	14:33	84 (Fig. S9h)	Shank3B$^{-/-}$	5-6	Male	Circular open field	White light
82	20200615	15:00	41 (Fig. S9h)	Shank3B$^{-/-}$	5-6	Male	Circular open field	White light
83	20200615	15:27	44 (Fig. S9h)	Shank3B$^{+/+}$	5-6	Male	Circular open field	White light
84	20200615	15:54	39 (Fig. S9h)	Shank3B$^{-/-}$	5-6	Male	Circular open field	White light
85	20200615	16:21	36 (Fig. S9h)	Shank3B$^{+/+}$	5-6	Male	Circular open field	White light

Supplementary Table 3 The individual level comparison between *Shank3B*$^{+/+}$ and *Shank3B*$^{-/-}$ mice

	KO1	KO2	KO3	KO4	KO5	KO6	KO7	KO8	KO9	KO10	Group Difference ▲
**WT1**	M5▽	M5▽	M5▽	M5▽	M5▽	M5▽	M5▽	M5▽	M5▽	M5▽	M5: 0
	M14▽	M14▽	M14▽	M14▽	M14▽	M14▲	M14▽	M14▽	M14▽	M14▲	M14: 2
	M21▽	M21▽	M21▽	M21▽	M21▽	M21▽	M21▽	M21▽	M21▽	M21▽	M21: 0
	M22▽	M22▽	M22▽	M22▽	M22▽	M22▽	M22▽	M22▽	M22▽	M22▽	M22: 0
	M38▲	M38▲	M38▲	M38▲	M38▲	M38▲	M38▲	M38▲	M38▲	M38▲	M38: 10
	M39▲	M39▲	M39▲	M39▲	M39▲	M39▲	M39▽	M39▲	M39▲	M39▲	M39: 9
	M40▲	M40▲	M40▲	M40▲	M40▲	M40▲	M40▲	M40▲	M40▲	M40▲	M40: 10
	M41▲	M41▲	M41▲	M41▲	M41▲	M41▲	M41▲	M41▲	M41▲	M41▲	M41: 10
**WT2**	M5▽	M5▽	M5▽	M5▽	M5▽	M5▽	M5▽	M5▽	M5▽	M5▽	M5: 0
	M14▽	M14▽	M14▽	M14▽	M14▽	M14▽	M14▽	M14▽	M14▽	M14▽	M14: 0
	M21▽	M21▽	M21▽	M21▽	M21▽	M21▽	M21▽	M21▽	M21▽	M21▽	M21: 0
	M22▽	M22▽	M22▽	M22▽	M22▽	M22▽	M22▽	M22▲	M22▽	M22▽	M22: 1
	M38▲	M38▲	M38▲	M38▲	M38▲	M38▽	M38▽	M38▲	M38▲	M38▲	M38: 8
	M39▲	M39▲	M39▲	M39▲	M39▲	M39▲	M39▲	M39▲	M39▲	M39▲	M39: 10
	M40▲	M40▲	M40▲	M40▲	M40▲	M40▲	M40▲	M40▲	M40▲	M40▲	M40: 10
	M41▲	M41▲	M41▲	M41▲	M41▲	M41▲	M41▲	M41▲	M41▲	M41▲	M41: 10
**WT3**	M5▽	M5▽	M5▽	M5▽	M5▽	M5▽	M5▽	M5▽	M5▽	M5▽	M5: 0
	M14▽	M14▽	M14▽	M14▽	M14▽	M14▽	M14▽	M14▽	M14▽	M14▽	M14: 0
	M21▽	M21▽	M21▽	M21▽	M21▽	M21▲	M21▽	M21▽	M21▽	M21▽	M21: 1
	M22▽	M22▽	M22▽	M22▽	M22▽	M22▽	M22▽	M22▽	M22▽	M22▽	M22: 0
	M38▲	M38▲	M38▲	M38▲	M38▲	M38▲	M38▽	M38▲	M38▲	M38▲	M38: 8
	M39▲	M39▲	M39▲	M39▲	M39▲	M39▲	M39▽	M39▲	M39▲	M39▲	M39: 9
	M40▲	M40▲	M40▲	M40▲	M40▲	M40▲	M40▲	M40▲	M40▲	M40▲	M40: 10
	M41▲	M41▲	M41▲	M41▲	M41▲	M41▲	M41▽	M41▲	M41▲	M41▲	M41: 9
**WT4**	M5▽	M5▽	M5▽	M5▽	M5▽	M5▽	M5▽	M5▽	M5▽	M5▽	M5: 0
	M14▽	M14▽	M14▽	M14▽	M14▽	M14▽	M14▽	M14▽	M14▽	M14▲	M14: 1
	M21▽	M21▽	M21▽	M21▽	M21▽	M21▽	M21▽	M21▽	M21▽	M21▽	M21: 0
	M22▽	M22▽	M22▽	M22▽	M22▽	M22▲	M22▲	M22▲	M22▽	M22▽	M22: 3
	M38▲	M38▲	M38▲	M38▲	M38▲	M38▲	M38▲	M38▲	M38▲	M38▲	M38: 10
	M39▲	M39▲	M39▲	M39▲	M39▲	M39▲	M39▲	M39▲	M39▲	M39▲	M39: 10
	M40▲	M40▲	M40▲	M40▲	M40▲	M40▲	M40▲	M40▲	M40▲	M40▲	M40: 10
	M41▲	M41▲	M41▲	M41▲	M41▲	M41▲	M41▲	M41▲	M41▲	M41▲	M41: 10
**WT5**	M5▽	M5▽	M5▽	M5▽	M5▽	M5▽	M5▽	M5▽	M5▽	M5▽	M5: 0
	M14▽	M14▽	M14▽	M14▽	M14▽	M14▽	M14▽	M14▽	M14▽	M14▽	M14: 0
	M21▽	M21▽	M21▽	M21▽	M21▽	M21▲	M21▲	M21▲	M21▲	M21▲	M21: 5
	M22▽	M22▽	M22▽	M22▽	M22▽	M22▲	M22▲	M22▲	M22▽	M22▽	M22: 3
	M38▲	M38▲	M38▲	M38▲	M38▲	M38▲	M38▲	M38▲	M38▲	M38▲	M38: 10
	M39▲	M39▲	M39▲	M39▲	M39▲	M39▲	M39▽	M39▲	M39▲	M39▲	M39: 9
	M40▲	M40▲	M40▲	M40▲	M40▲	M40▲	M40▽	M40▲	M40▲	M40▲	M40: 9
	M41▲	M41▲	M41▲	M41▲	M41▲	M41▲	M41▽	M41▲	M41▲	M41▲	M41: 9

**WT6**	M5▽	M5▽	M5▽	M5▽	M5▲	M5▽	M5▽	M5▽	M5▲	M5▽	M5: 2
	M14▽	M14▽	M14▽	M14▽	M14▲	M14▲	M14▽	M14▲	M14▽	M14▲	M14: 4
	M21▽	M21▽	M21▽	M21▽	M21▽	M21▲	M21▽	M21▲	M21▲	M21▽	M21: 3
	M22▽	M22▽	M22▽	M22▽	M22▽	M22▽	M22▽	M22▲	M22▽	M22▽	M22: 1
	M38▲	M38▲	M38▲	M38▲	M38▲	M38▲	M38▲	M38▲	M38▲	M38▲	M38: 10
	M39▲	M39▲	M39▲	M39▲	M39▲	M39▲	M39▲	M39▲	M39▲	M39▲	M39: 10
	M40▲	M40▲	M40▲	M40▲	M40▲	M40▲	M40▽	M40▲	M40▽	M40▲	M40: 8
	M41▲	M41▲	M41▲	M41▲	M41▲	M41▲	M41▽	M41▽	M41▲	M41▲	M41: 8
**WT7**	M5▽	M5▽	M5▽	M5▽	M5▽	M5▽	M5▽	M5▽	M5▽	M5▽	M5: 0
	M14▽	M14▽	M14▽	M14▽	M14▽	M14▲	M14▽	M14▽	M14▽	M14▲	M14: 2
	M21▽	M21▽	M21▽	M21▽	M21▽	M21▲	M21▽	M21▽	M21▽	M21▽	M21: 1
	M22▽	M22▽	M22▽	M22▽	M22▽	M22▽	M22▽	M22▽	M22▽	M22▽	M22: 0
	M38▲	M38▲	M38▲	M38▲	M38▲	M38▽	M38▽	M38▲	M38▲	M38▲	M38: 8
	M39▲	M39▲	M39▲	M39▲	M39▲	M39▲	M39▽	M39▲	M39▲	M39▲	M39: 9
	M40▲	M40▲	M40▲	M40▲	M40▲	M40▲	M40▽	M40▲	M40▲	M40▲	M40: 9
	M41▲	M41▲	M41▲	M41▲	M41▲	M41▲	M41▲	M41▲	M41▲	M41▲	M41: 10
**WT8**	M5▽	M5▲	M5▽	M5▽	M5▲	M5▲	M5▲	M5▽	M5▲	M5▲	M5: 6
	M14▽	M14▽	M14▽	M14▽	M14▲	M14▲	M14▽	M14▲	M14▽	M14▲	M14: 4
	M21▽	M21▽	M21▽	M21▽	M21▽	M21▲	M21▽	M21▽	M21▽	M21▽	M21: 1
	M22▽	M22▽	M22▽	M22▽	M22▽	M22▲	M22▽	M22▲	M22▽	M22▽	M22: 3
	M38▲	M38▲	M38▲	M38▲	M38▲	M38▽	M38▽	M38▲	M38▽	M38▲	M38: 7
	M39▲	M39▲	M39▲	M39▲	M39▲	M39▽	M39▽	M39▲	M39▲	M39▲	M39: 8
	M40▲	M40▲	M40▲	M40▲	M40▲	M40▲	M40▽	M40▲	M40▲	M40▲	M40: 9
	M41▲	M41▲	M41▲	M41▲	M41▲	M41▲	M41▲	M41▲	M41▲	M41▲	M41: 10
**WT9**	M5▽	M5▽	M5▽	M5▽	M5▽	M5▽	M5▽	M5▽	M5▽	M5▽	M5: 0
	M14▽	M14▽	M14▽	M14▽	M14▽	M14▽	M14▽	M14▽	M14▽	M14▲	M14: 1
	M21▽	M21▽	M21▽	M21▽	M21▽	M21▲	M21▽	M21▽	M21▽	M21▽	M21: 1
	M22▽	M22▽	M22▽	M22▽	M22▽	M22▲	M22▲	M22▲	M22▽	M22▽	M22: 3
	M38▲	M38▲	M38▲	M38▲	M38▲	M38▽	M38▽	M38▲	M38▽	M38▲	M38: 7
	M39▲	M39▲	M39▲	M39▲	M39▲	M39▲	M39▽	M39▲	M39▲	M39▲	M39: 9
	M40▲	M40▲	M40▲	M40▲	M40▲	M40▲	M40▲	M40▲	M40▲	M40▲	M40: 10
	M41▲	M41▲	M41▲	M41▲	M41▲	M41▲	M41▲	M41▲	M41▲	M41▲	M41: 10
**WT10**	M5▽	M5▽	M5▽	M5▽	M5▽	M5▽	M5▽	M5▽	M5▽	M5▽	M5: 0
	M14▽	M14▽	M14▽	M14▽	M14▽	M14▽	M14▽	M14▽	M14▽	M14▽	M14: 0
	M21▽	M21▽	M21▽	M21▽	M21▽	M21▲	M21▽	M21▽	M21▽	M21▽	M21: 1
	M22▽	M22▽	M22▽	M22▽	M22▽	M22▽	M22▽	M22▽	M22▽	M22▽	M22: 0
	M38▲	M38▲	M38▲	M38▲	M38▲	M38▲	M38▽	M38▲	M38▲	M38▲	M38: 9
	M39▲	M39▲	M39▲	M39▲	M39▲	M39▲	M39▲	M39▲	M39▲	M39▲	M39: 10
	M40▲	M40▲	M40▲	M40▲	M40▲	M40▲	M40▽	M40▲	M40▲	M40▲	M40: 9
	M41▲	M41▲	M41▲	M41▲	M41▲	M41▲	M41▲	M41▲	M41▲	M41▲	M41: 10
**All Group Difference▲**	M5: 8, M14: 14, M21: 13, M22: 14, M38: 87, M39: 93, M40: 94, M41: 96										

M*X*: *X* the index of behavioral module;

▲: KO individual shows higher fraction of *X* behavioral module than WT;

▽: KO individual shows lower fraction of *X* behavioral module than WT;

Group Difference ▲(M*X*: *n*): The number of KO individuals with higher *X* behavior modulus fractions than WT is n;

All Group Difference ▲(MX: *m*): In all the 100 times paired-wise comparisons, the number of times that KO individuals with higher *X* behavior modulus fractions than WT is m.

Reference

1. Zhang, Z. & Member, S. A Flexible New Technique.pdf. *IEEE Transactions on Pattern Analysis and Machine Intelligence* **22**, 1330–1334 (2000).

2. Mathis, A. *et al.* DeepLabCut: markerless pose estimation of user-defined body parts with deep learning. *Nature Neuroscience* **21**, 1281–1289 (2018).

3. Wilkinson, J. H., Reinsch, C., Golub, G. H. & Reinsch, C. Singular Value Decomposition and Least Squares Solutions. in *Linear Algebra* 134–151 (Springer Berlin Heidelberg, 1971). doi:10.1007/978-3-662-39778-7_10.

4. Delaunay, B. & others. Sur la sphere vide. *Izv Akad Nauk SSSR, Otdelenie Matematicheskii i Estestvennyka Nauk* **7**, 1–2 (1934).

5. Gong, Y. *et al.* High-speed recording of neural spikes in awake mice and flies with a fluorescent voltage sensor. *Science* **350**, 1361–1366 (2015).

6. Haber, S. B. & Simmel, E. C. Tail rattling and agonistic behavior in mice: Coincidental or causal? *Bulletin of the Psychonomic Society* **7**, 84–86 (1976).

7. McInnes, L., Healy, J. & Melville, J. UMAP: Uniform Manifold Approximation and Projection for Dimension Reduction. (2018).

8. Keerthi, S. S. & Lin, C. J. Asymptotic behaviors of support vector machines with gaussian kernel. *Neural Computation* **15**, 1667–1689 (2003).

9. Zhou, F., Member, S., Torre, F. De & Hodgins, J. K. for Temporal Clustering of Human Motion. *Ieee Transactions on Pattern Analysis and Machine Intelligence* **35**, 1–15 (2013).

10. Zhou, F., De La Torre, F. & Hodgins, J. K. Aligned cluster analysis for temporal segmentation of human motion. *2008 8th IEEE International Conference on Automatic Face and Gesture Recognition, FG 2008* (2008) doi:10.1109/AFGR.2008.4813468.

11. Von Luxburg, U. A tutorial on spectral clustering. *Statistics and Computing* **17**, 395–416 (2007).

12. Cheadle, C., Vawter, M. P., Freed, W. J. & Becker, K. G. Analysis of microarray data using Z score transformation. *Journal of Molecular Diagnostics* **5**, 73–81 (2003).

13. Johnson, S. C. Hierarchical clustering schemes. *Psychometrika* **32**, 241–254 (1967).

14. Corpet, F. Multiple sequence alignment with hierarchical clustering. *Nucleic Acids Research* **16**, 10881–10890 (1988).

15. Chomboon, K., Chujai, P., Teerarassammee, P., Kerdprasop, K. & Kerdprasop, N. An Empirical Study of Distance Metrics for k-Nearest Neighbor Algorithm. 280–285 (2015) doi:10.12792/iciae2015.051.

16. Critchley, F. On certain linear mappings between inner-product and squared-distance matrices. *Linear Algebra and Its Applications* **105**, 91–107 (1988).

17. Stringer, C. *et al.* Spontaneous behaviors drive multidimensional, brainwide activity. *Science* **364**, (2019).

18. Orlova, D. Y. *et al.* QFMatch: Multidimensional flow and mass cytometry samples alignment. *Scientific Reports* **8**, 1–14 (2018).

19. Wang, X. *et al.* Synaptic dysfunction and abnormal behaviors in mice lacking major isoforms of Shank3. *Human Molecular Genetics* **20**, 3093–3108 (2011).

20. Aitta-aho, T., Pappa, E., Burdakov, D. & Apergis-Schoute, J. Cellular activation of hypothalamic hypocretin/orexin neurons facilitates short-term spatial memory in mice. *Neurobiology of Learning and Memory* **136**, 183–188 (2016).

21. Liu, D. *et al.* A common hub for sleep and motor control in the substantia nigra. *Science* **367**, 440–445 (2020).

22. Wiltschko, A. B. *et al.* Mapping Sub-Second Structure in Mouse Behavior. *Neuron* **88**, 1121–1135 (2015).

23. Markowitz, J. E. *et al.* The Striatum Organizes 3D Behavior via Moment-to-Moment Action Selection. *Cell* **174**, 44-58.e17 (2018).
