# Supplementary material for: A hierarchical 3D-motion learning framework for animal spontaneous behavior mapping
Source: Nat Commun. 2021 May 13;12:2784. doi: 10.1038/s41467-021-22970-y (PMC8119960; doi:10.1038/s41467-021-22970-y)
Supplement: Supplementary file 2 — Reporting Summary [file 41467_2021_22970_MOESM2_ESM.pdf]

nature research

Corresponding author(s): Liping Wang

Last updated by author(s): Mar 30, 2021

Reporting Summary

Nature Research wishes to improve the reproducibility of the work that we publish. This form provides structure for consistency and transparency in reporting. For further information on Nature Research policies, see our Editorial Policies and the Editorial Policy Checklist.

Statistics

For all statistical analyses, confirm that the following items are present in the figure legend, table legend, main text, or Methods section.

n/a	Confirmed	
☐	☒	The exact sample size (n) for each experimental group/condition, given as a discrete number and unit of measurement
☐	☒	A statement on whether measurements were taken from distinct samples or whether the same sample was measured repeatedly
☐	☒	The statistical test(s) used AND whether they are one- or two-sided<br>*Only common tests should be described solely by name; describe more complex techniques in the Methods section.*
☒	☐	A description of all covariates tested
☐	☒	A description of any assumptions or corrections, such as tests of normality and adjustment for multiple comparisons
☐	☒	A full description of the statistical parameters including central tendency (e.g. means) or other basic estimates (e.g. regression coefficient) AND variation (e.g. standard deviation) or associated estimates of uncertainty (e.g. confidence intervals)
☐	☒	For null hypothesis testing, the test statistic (e.g. F, t, r) with confidence intervals, effect sizes, degrees of freedom and P value noted<br>*Give P values as exact values whenever suitable.*
☒	☐	For Bayesian analysis, information on the choice of priors and Markov chain Monte Carlo settings
☒	☐	For hierarchical and complex designs, identification of the appropriate level for tests and full reporting of outcomes
☒	☐	Estimates of effect sizes (e.g. Cohen's d, Pearson's r), indicating how they were calculated

Our web collection on statistics for biologists contains articles on many of the points above.

Software and code

Policy information about availability of computer code

Data collection	Multi-view behavior video streams collection and calibration checkerboard images acquisition: custom codes written in Python (3.7.x) that relies on opencv-python (3.4.9.33) and pyrealsense2 (2.26.0.1053); Camera calibration: 3D calibration with MATLAB's StereoCameraCalibrator GUI; 3D skeleton reconstruction: pose3d (Sheshadri, 2020); Pose estimation: DeepLabCut (2.1.6.4).
Data analysis	3D behavioral trajectories decomposition: the open-source toolbox Behavior Atlas (can be accessed at https://behavioratlas.tech/ and are available on Zenodo: https://doi.org/10.5281/zenodo.4626951.) relies on third-party package aca (Zhou F., 2013) and UMAP (1.5.x); Statistics: Prism 8.0 (GraphPad Software); Other data analysis are based on custom codes written in MATLAB (2020).

For manuscripts utilizing custom algorithms or software that are central to the research but not yet described in published literature, software must be made available to editors and reviewers. We strongly encourage code deposition in a community repository (e.g. GitHub). See the Nature Research guidelines for submitting code & software for further information.

Data

Policy information about availability of data

All manuscripts must include a data availability statement. This statement should provide the following information, where applicable:

- Accession codes, unique identifiers, or web links for publicly available datasets
- A list of figures that have associated raw data
- A description of any restrictions on data availability

All the raw videos and 3D skeleton trajectories associated with Shank3B-/- mice spontaneous behavior test showing in Fig. 6 are available in the Zenodo repository https://doi.org/10.5281/zenodo.4629544 and Supplementary information. Any other relevant data are available upon reasonable request. Source data are provided with this paper.

Field-specific reporting

Please select the one below that is the best fit for your research. If you are not sure, read the appropriate sections before making your selection.

☒ Life sciences ☐ Behavioural & social sciences ☐ Ecological, evolutionary & environmental sciences

For a reference copy of the document with all sections, see nature.com/documents/nr-reporting-summary-flat.pdf

Life sciences study design

All studies must disclose on these points even when the disclosure is negative.

Sample size

Fig. 6: Total 20 young adult (5-6 weeks) male Shank3B-/- (n=10) and Shank3B+/+ (n=10) mice with C57BL/6J genetic background were used in the behavioral experiment.

Supplementary Fig. 12: Total 84 behavioral data which include: 5-6 week old, male mice in white light in the circular open-field test, 10 samples in each group (KO1 and WT1); We added new groups according to different experimental apparatus (KO2 and WT2), lighting conditions (KO3 and WT3), ages (KO4 and WT4), and sexes (KO5 and WT5), 8 KO and 8 WT mice for each condition.

Supplementary Fig. 13: Total one sample used for 24 hours behavior monitoring. The male mouse used in this experiment has C57BL/6J genetic background and is 13 weeks old.

The samples description is listed in Supplementary Table 2.

No statistical methods were used to pre-determine sample sizes, and our sample sizes were selected by referring to the previous related studies (Peça, J. et al., Nature, 2011; Mei Y. et al., Nature, 2016; Wang X. et al., Human Molecular Genetics, 2011; Fourie, C. et al., Frontiers in Cellular Neuroscience, 2018.) and verified by power analysis (Rosenthal, R. et al., The handbook of research synthesis, 1994).

Data exclusions

No data was excluded.

Replication

To verify whether our framework can identify the behavioral biomarkers of the animal disease model. We performed the open-field behavioral test with n=10 biological replicates for each genotype of mice. All mice were tested once, and no individual replicate was excluded. All attempts at replication were successful.

Randomization

Before the open field test, the Shank3B-/- (n=10) and Shank3B+/+ (n=10) mice were mixed then housed in three cages. We performed the behavior test of each mouse in a random order.

Blinding

All behavioral experiments and preliminary analysis were performed and analyzed blinded to genotypes. Experimenters were not blind to group during the analysis of spontaneous behavior of Shank3B-/- and WT mice to identify the behavioral biomarker of Shank3B-/-.

Reporting for specific materials, systems and methods

We require information from authors about some types of materials, experimental systems and methods used in many studies. Here, indicate whether each material, system or method listed is relevant to your study. If you are not sure if a list item applies to your research, read the appropriate section before selecting a response.

Materials & experimental systems

n/a	Involved in the study	
☒	☐	Antibodies
☒	☐	Eukaryotic cell lines
☒	☐	Palaeontology and archaeology
☐	☒	Animals and other organisms
☒	☐	Human research participants
☒	☐	Clinical data
☒	☐	Dual use research of concern

Methods

n/a	Involved in the study	
☒	☐	ChIP-seq
☒	☐	Flow cytometry
☒	☐	MRI-based neuroimaging

Animals and other organisms

Policy information about studies involving animals; ARRIVE guidelines recommended for reporting animal research

Laboratory animals

Shank3B-/- mice were obtained from the Jackson Laboratory (Jax No. 017688). Shank3B-/- and Shank3B+/+ mouse lines have been described in previous study (Peça, J. et al, Nature, 2011).

The mice were housed at 4–6 mice per cage under a 12-h light-dark cycle at 22–25°C with 40–70% humidity, and were allowed to

	access water and food ad libitum.
Wild animals	This study did not involve wild animals.
Field-collected samples	This study did not involve samples collected from the field.
Ethics oversight	All husbandry and experimental procedures in this study were approved by Animal Care and Use Committees at the Shenzhen Institute of Advanced Technology (SIAT), Chinese Academy of Sciences (CAS).

Note that full information on the approval of the study protocol must also be provided in the manuscript.
