# Supplementary material for: A hierarchical 3D-motion learning framework for animal spontaneous behavior mapping
Source: Nat Commun. 2021 May 13;12:2784. doi: 10.1038/s41467-021-22970-y (PMC8119960; doi:10.1038/s41467-021-22970-y)
Supplement: Supplementary file 11 — Description of Additional Supplementary Files [file 41467_2021_22970_MOESM11_ESM.pdf]

Title: Supplementary Movie 1
Description: Demonstration of four-view mouse video capturing and 3D tracking

Title: Supplementary Movie 2
Description: Demonstration of behavior decomposition

Title: Supplementary Movie 3
Description: Demonstration of behavior mapping with low-dimensionality embedding

Title: Supplementary Movie 4
Description: Test in square open field

Title: Supplementary Movie 5
Description: Test in sociability cage

Title: Supplementary Movie 6
Description: 3D tracking 2 mice with different appearances

Title: Supplementary Movie 7
Description: 3D tracking 2 mice with similar appearances
